# Utilizing Multi-Omics Analysis to Elucidate the Molecular Mechanisms of Oat Responses to Drought Stress

**DOI:** 10.3390/plants14050792

**Published:** 2025-03-04

**Authors:** Xiaojing Chen, Jinghui Liu, Baoping Zhao, Junzhen Mi, Zhongshan Xu

**Affiliations:** 1 National Outstanding Talents in Agricultural Research and Their Innovative Teams, Hohhot 010019, China; 13214088889@163.com (X.C.); cauljh@aliyun.com (J.L.); zhaobaoping82@163.com (B.Z.); mijunling1206@126.com (J.M.); 2 Cereal Engineering Technology Research Center, Inner Mongolia Autonomous Region, Hohhot 010019, China; 3 College of Life Sciences, Inner Mongolia Agricultural University, Hohhot 010019, China

**Keywords:** oat, drought stress, transcriptomics, proteomics, multi-omics

## Abstract

The oat is a crop and forage species with rich nutritional value, capable of adapting to various harsh growing environments, including dry and poor soils. It plays an important role in agricultural production and sustainable development. However, the molecular mechanisms underlying the responses of oat to drought stress remain unclear, warranting further research. In this study, we conducted a pot experiment with the drought-resistant cultivar JiaYan 2 (JIA2) and water-sensitive cultivar BaYou 9 (BA9) during the booting stage under three water gradient treatment conditions: 30% field capacity (severe stress), 45% field capacity (moderate stress), and 70% field capacity (normal water supply). After 7 days of stress, root samples were collected for transcriptome and proteome analyses. Transcriptome analysis revealed that under moderate stress, JIA2 upregulated 1086 differential genes and downregulated 2919 differential genes, while under severe stress, it upregulated 1792 differential genes and downregulated 4729 differential genes. Under moderate stress, BA9 exhibited an upregulation of 395 differential genes, a downregulation of 669, and an upregulation of 886 differential genes, and it exhibited 439 downregulations under severe stress. Under drought stress, most of the differentially expressed genes (DEGs) specific to JIA2 were downregulated, mainly involving redox reactions, carbohydrate metabolism, plant hormone signal regulation, and secondary metabolism. Proteomic analysis revealed that in JIA2, under moderate stress, 489 differential proteins were upregulated and 394 were downregulated, while 493 differential proteins were upregulated and 701 were downregulated under severe stress. In BA9, 590 and 397 differential proteins were upregulated under moderate stress, with 126 and 75 upregulated differential proteins under severe stress. Correlation analysis between transcriptomics and proteomics demonstrated that compared with no drought stress, four types of differentially expressed proteins (DEPs) were identified in the JIA2 differential gene–protein interaction network analysis under severe stress. These included 13 key cor DEGs and DEPs related to plant hormone signal transduction, biosynthesis of secondary metabolites, carbohydrate metabolism processes, and metabolic pathways. The consistency of gene and protein expression was validated using qRT-PCR, indicating their key roles in the strong drought resistance of JIA2.

## 1. Introduction

Under natural conditions, plant growth is often influenced by abiotic stresses such as drought, salinity, high temperatures, and cold. Enhancing crop stress resistance and developing new cultivars with improved tolerance and yield potential currently constitute the most economical strategy for boosting agricultural productivity. Among these stresses, drought is one of the most detrimental environmental factors affecting plant growth and development. With the escalating impacts of global climate change, drought has become a global challenge that restricts plant productivity [1,2,3].

The inhibition of growth and development is one of the most evident plant responses to drought stress. Drought can result in reduced plant height, fewer nodes, decreased leaf area, lower dry matter accumulation, prolonged growth periods, and significantly reduced yields [4,5,6]. In response to drought, plants accumulate substantial amounts of organic compounds and inorganic ions to increase cell fluid concentrations, lower osmotic potential, enhance water retention, maintain cellular structure and the spatial arrangement of biomacromolecules, and promote root growth under severe water-deficient conditions [7,8].

Proteomics and transcriptomics are extensively used to investigate plant responses to salt and drought stresses [9,10]. For example, the specifically or commonly regulated DEGs and DEP genes involved in heat shock protein biosynthesis, secondary metabolism biosynthesis, and hormone biosynthesis play a crucial role in cassava’s resistance to drought stress [11]. Under drought stress, plant cells sense and process stress signals through signal sensors, converting extracellular signals into intracellular signals. These signals are transmitted through distinct transduction pathways, where plant hormones, secondary messengers, signal transducers, and transcriptional regulators play important roles [12]. Transcription factor families such as WRKY, NAC, bZIP, AP2/EREBP, and MYB are closely associated with gene expression regulation under water deficit conditions [13].

Oat (*Avena sativa* L.) is classified as a whole grain rich in nutrients such as beta-glucan, fat, protein, minerals, and polyphenols [14]. As a dual-purpose crop for grain and feed, it exhibits drought and barrenness tolerance, making it well suited for cultivation in arid and semi-arid regions. As an advantageous characteristic crop in Inner Mongolia, its planting area and total yield rank first in China, playing a significant role in the development of local agriculture and animal husbandry [15]. Drought inhibits oat growth, leading to reductions in plant height, dry matter accumulation rate, root length, area, and volume compared to conditions involving an adequate water supply [16]. To cope with drought, plants accumulate various organic and inorganic substances within cells to maintain the osmotic balance between intracellular and extracellular environments [17]. However, drought stress can increase osmotic pressure in oat cells, disrupting normal growth. Additionally, it lowers the net photosynthetic rate, the transpiration rate, and stomatal conductance in oat leaves. Intercellular CO_2_ concentrations decrease under mild drought stress but increase under moderate and severe drought stress. In one instance, during the critical period of drought stress, light, moderate, and severe drought led to yield reductions ranging from 9.5% to 12.7%, 16.8% to 27.0%, and 44.1% to 47.7%, respectively [18]. Under drought stress, the auxin, cytokinin, and brassinosteroid signaling pathways in Longyan 3 are inhibited, while the abscisic and jasmonic acid signaling pathways are activated. Upregulation of genes such as PP2C (CL3379. Contig7_All), ABF (Unigene18890_All), SNRK2 (CL10999. Contig3_All), GID1 (Unigene13457_All), and MYC2 (CL1579. Contig1_All) may enhance drought resistance in DA92-2F6 [19].

Currently, research on oat responses to drought stress primarily focuses on growth, physiological and biochemical indicators, and single-omics studies (transcriptomics, proteomics, or metabolomics). Multi-omics association analysis has been applied to investigate oat responses to salt or phosphorus stress. However, no studies have used multi-omics association analysis to explore the drought resistance mechanism in the oat [20]. Therefore, building on previous research carried out by our group, in this study, we selected oat cultivars with varying drought resistances and employed a combination of transcriptomics and proteomics to identify differentially expressed genes (DEGs) linked to drought resistance. We also aimed to explore key metabolic pathways involved in oat responses to drought stress, providing a theoretical foundation and technical reference for breeding drought-resistant oat germplasms and advancing high-yield-cultivation-technology research.

## 2. Results

### 2.1. Effects of Drought Stress on Growth of Two Oat Cultivars

**Figure 1:**
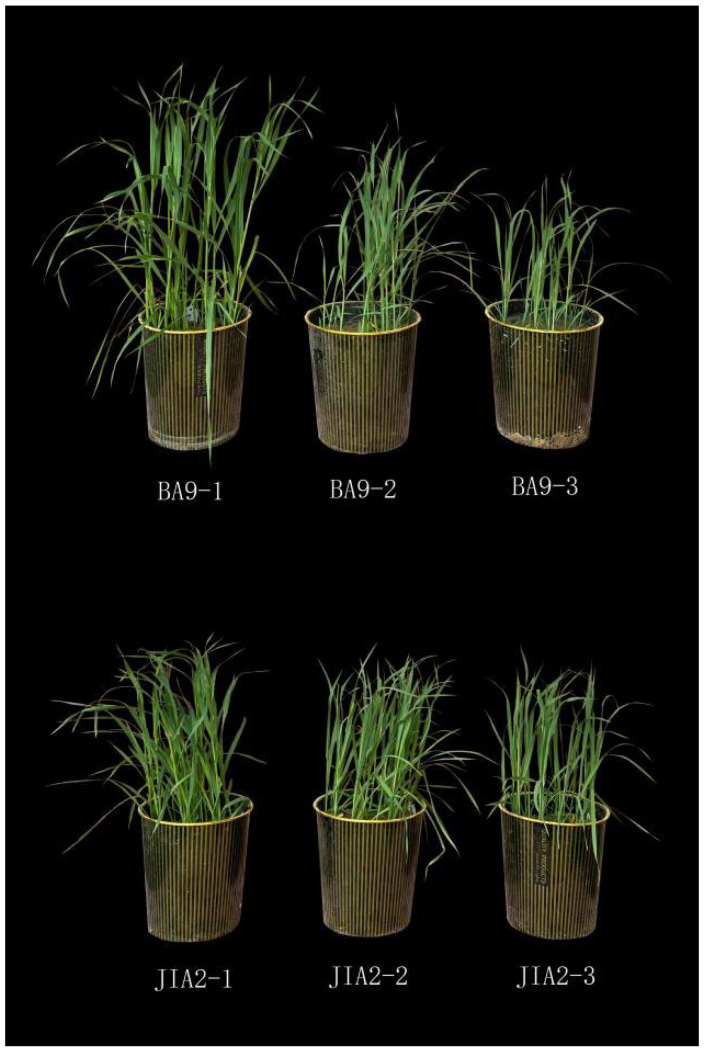
Differences in growth morphologies of two oat cultivars with different drought tolerances after being subjected to drought stress. BA9-1, BA9-2, BA9-3, JIA2-1, JIA2-2, and JIA2-3 represent the normal water supply (70%), moderate drought stress (45%), and severe drought stress (30%) conditions for BA9 and JIA 2, respectively.

The growth of both cultivars was negatively affected by drought stress during the seedling stage. Under drought stress, BA9 exhibited greater growth inhibition compared with JIA2, with BA9-1 demonstrating significantly better growth than BA9-2 and BA9-3 (Figure 1).

### 2.2. Transcriptome-Sequencing-Data Quality Control and Transcriptome Analysis

**Figure 2:**
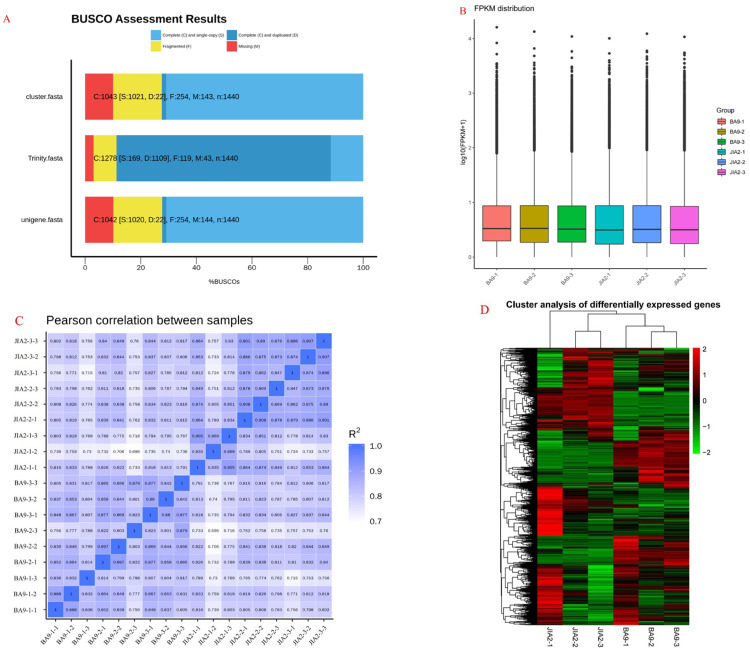
Sequencing sample evaluation of transcriptomics of two oat cultivars with different drought tolerances under drought stress. Note: (**A**) BUSCO assessment completeness statistical chart. (**B**) A boxplot figure depicting FPKM under different experimental conditions. The *X*-axis represents different conditions, and the *Y*-axis is the log10 (FPKM) value of different genes. (**C**) A heatmap of correlation coefficient for the correlation between samples. (**D**) Genes that are differentially expressed in BA9 and JIA2 in response to drought stress. BA9-1, BA9-2, BA9-3, JIA2-1, JIA2-2, and JIA2-3 represent the normal water supply (70%), moderate drought stress (45%), and severe drought stress (30%) conditions for BA9 and JIA 2, respectively.

Comparative transcriptomic sequencing of the oat roots was performed to reveal the molecular mechanisms and identify the key genes involved in the responses of oat seedlings with different drought resistances to drought stress. We collected samples of BA9 and JIA2 subjected to moderate and severe stress for transcriptomic analysis after 0 and 7 days. By assembling the complete BUSCO gene RNA-Seq reads from scratch, the mapping rate reached 88.75% (Figure 2A). Differential expression analysis of inter-sample data was performed using FPKM (Figure 2B), with a high correlation observed between the biological replicates of each treatment (Figure 2C). Based on these data, we can infer that the biological replicates of the sequencing samples are reliable, and a significant difference exists between drought stress and no drought stress (Figure 2D).

**Figure 3:**
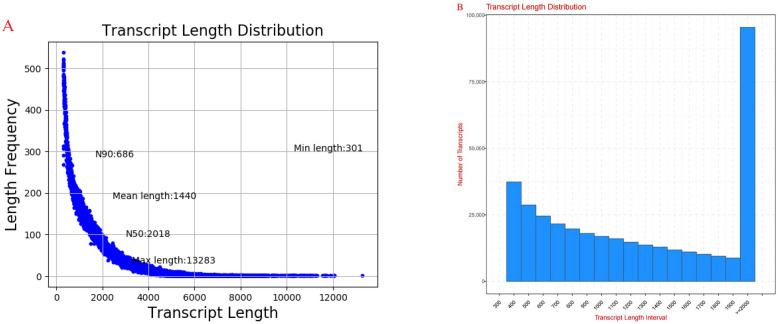
Transcript length distribution of two oat cultivars with different drought tolerances under drought stress. Note: (**A**) Transcript Length Distribution. The horizontal axis in the figure represents transcript Length, and the vertical axis represents length frequency. (**B**) Gene sequence length distribution map. The horizontal axis in the figure represents gene length/length interval, and the vertical axis represents the number of genes.

This sequencing process generated a total of 393,328 reads, with a total length of 566,345,053 bp. The longest sample was 13,283 bp, with an average length of 1440 bp (Figure 3A). Sequence length analysis of the 393,328 transcripts revealed that 76.75% (301,870) of the sequences were below 2000 bp in length. Specifically, there were 75,207 sequences under 500 bp long, 105,908 between 500 and 1000 bp long, 120,755 between 1000 and 2000 bp long, and 91,458 sequences exceeding 2000 bp in length (Figure 3B). Based on annotation results from the Nr library, species distribution maps on the comparison were statistically analyzed and plotted. The results indicated that the top species identified in the comparison were barley, wheat, Ural wheat, rice, and corn.

### 2.3. Annotations of Transcription Factors in Oat Roots with Different Drought Resistances Subjected to Drought Stress

**Figure 4:**
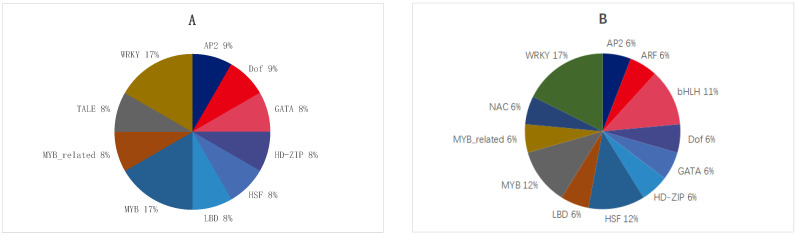
Differentially expressed transcription factors of JIA2 under drought stress. Note: (**A**) represents moderate drought stress treatment. (**B**) represents severe drought stress treatment.

Under moderate stress, JIA2′s differentially expressed transcription factors were classified into 10 categories, including 2 in MYB, 2 in WRKY, 1 in AP2, 1 in GATA, and 1 in HSF (Figure 4A). Under severe stress, JIA2′s differentially expressed transcription factors were classified into 12 categories, including 3 in WRKY, 2 in MYB, 2 in HSF, 2 in bHLH, and 1 in GATA (Figure 4B).

Under moderate stress, no DEGs in BA9 were identified when compared with the Arabidopsis transcription factor database. Under moderate stress, three upregulated and nine downregulated DEGs in JIA2 were found when compared with the Arabidopsis transcription factor database (Table 1). Under severe stress, JIA2 exhibited 5 upregulated and 12 downregulated DEGs in comparison with the Arabidopsis transcription factor database (Table 2).

### 2.4. Identification of DEGs in Oat Cultivars with Different Drought Resistances Under Drought Stress

**Figure 5:**
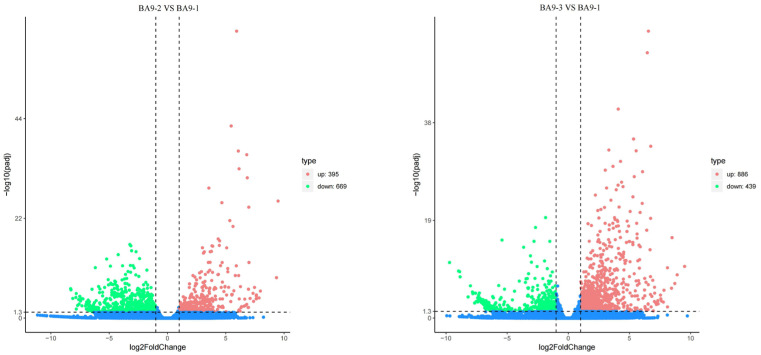

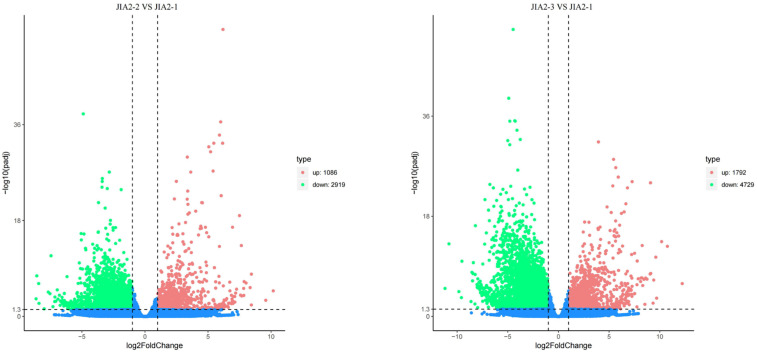
The number of differentially expressed genes in two oat cultivars under drought stress. Note: Red represents upregulation, green represents downregulation, and blue represents insignificant difference. Note: BA9-1, BA9-2, BA9-3, JIA2-1, JIA2-2, and JIA2-3 represent the normal water supply (70%), moderate drought stress (45%), and severe drought stress (30%) conditions for BA9 and JIA 2, respectively.

Under moderate stress, BA9 exhibited 395 upregulated and 669 downregulated genes, while under severe stress, BA9 exhibited 886 upregulated and 439 downregulated genes. Under moderate stress, JIA2 exhibited 1086 upregulated and 2919 downregulated genes, while under severe stress, JIA2 exhibited 1792 upregulated and 4729 downregulated genes (Figure 5).

As presented in Table S1, the expression trends of the same DEGs under moderate and severe stress were consistent for BA9 and JIA2. Under the same stress conditions, the expression trends of the same genes in BA9 and JIA2 remained consistent. Table S2 lists the DEGs in JIA2 under drought stress. Under drought stress, the expression trends of the same genes were consistent, with most DEGs being downregulated, mainly those related to redox reactions, carbohydrate metabolism, plant-growth-hormone-signaling regulation, and secondary metabolism.

### 2.5. GO and KEGG Enrichment Analyses of DEGs

**Figure 6:**
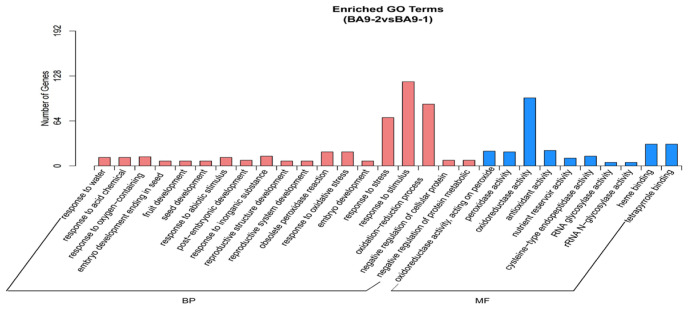

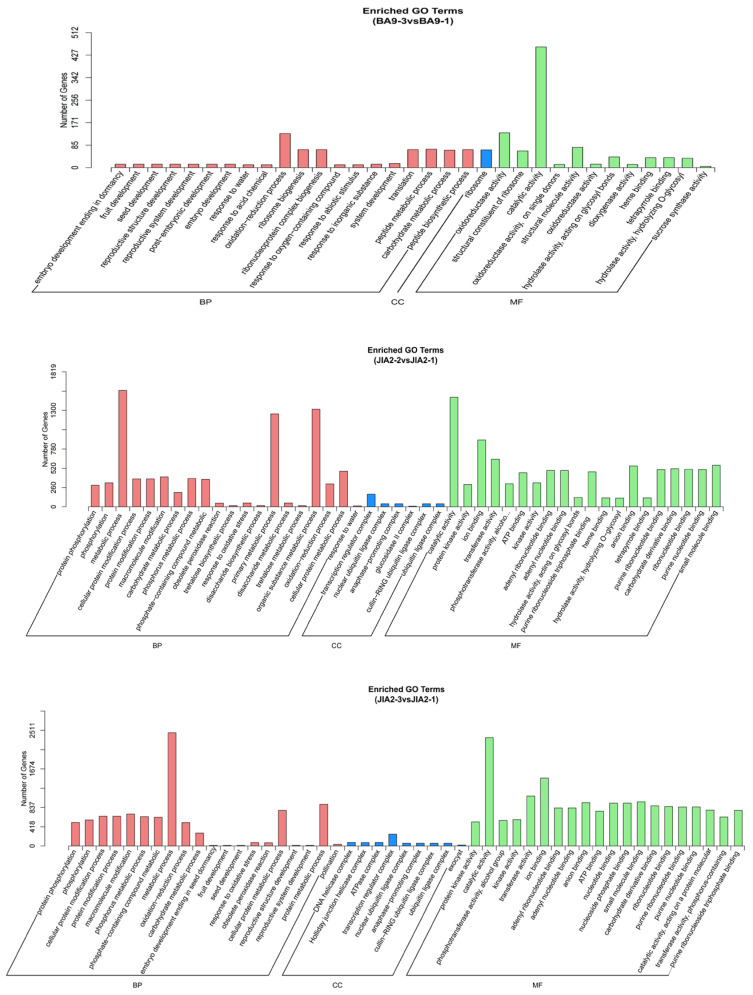
Analysis of GO enrichment of differentially expressed genes of two oat cultivars with different drought tolerances under drought stress. Note: BA9-1, BA9-2, BA9-3, JIA2-1, JIA2-2, and JIA2-3 represent the normal water supply (70%), moderate drought stress (45%), and severe drought stress (30%) conditions for BA9 and JIA 2, respectively.

GO classification of DEGs indicated that, under moderate stress, BA9 genes were mainly enriched in response to stimuli (120), oxidoreductase activity (97), redox processes (88), and response to stress (88). Under severe stress, BA9 genes were mainly enriched in catalytic activity (458), oxidoreductase activity (132), redox processes (129), structural molecular activity (77), peptide metabolism processes (70), and ribosomes (67). For JIA2, under moderate stress, DEGs were mainly enriched in metabolic processes (1569), catalytic activity (1476), organic metabolism processes (1315), and primary metabolism processes (1252). Under severe stress, JIA2 genes were mainly enriched in metabolic processes (2460), catalytic activity (2352), ion binding (1475), and transferase activity (1085). Furthermore, under drought stress, BA9 exhibited the most pronounced changes in redox activity and processes, while JIA2 demonstrated the most significant changes in metabolic processes (Figure 6).

**Figure 7:**
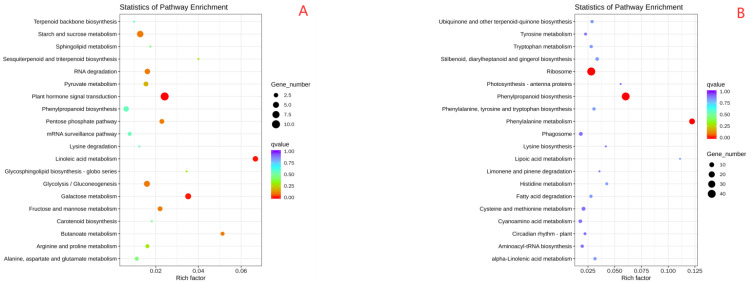

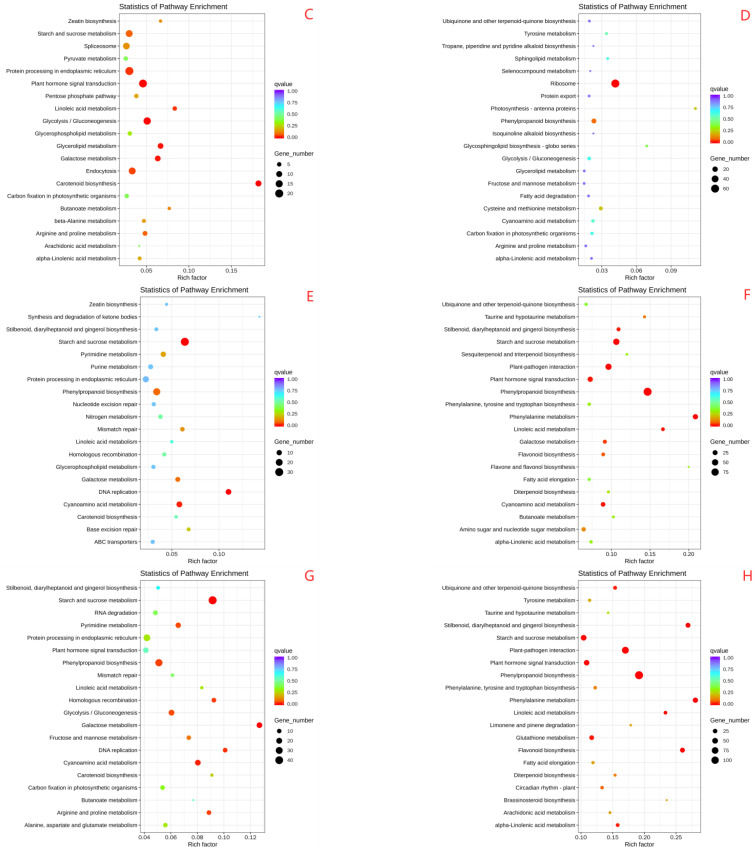
Analysis of KEGG enrichment of differentially expressed genes of two oat cultivars with different drought tolerances under drought stress. Note: (**A**) represents BA9-2 treatment upregulated genes; (**B**) represents BA9-2 treatment downregulated genes; (**C**) represents BA9-3 treatment upregulated genes; (**D**) represents BA9-3 treatment downregulated genes; (**E**) represents JIA2-2 treatment upregulated genes; (**F**) represents JIA2-2 treatment downregulated genes; (**G**) represents JIA2-3 treatment upregulated genes; (**H**) represents JIA2-3 treatment downregulated genes.

The KEGG database was used to identify the primary metabolic pathways associated with the DEGs (Figure 7). Under moderate stress, the co-upregulated genes in BA9 and JIA2 were predominantly involved in starch and sucrose metabolism (6/30, with the numbers representing the genes in the BA9 and JIA2 pathways, respectively), phenylpropane biosynthesis (4/22), galactose metabolism (5/8), carotenoid biosynthesis (1/3), and linoleic acid metabolism (4/3). The pathways uniquely upregulated in BA9 genes under moderate stress included plant hormone signal transduction (10), glycolysis (5), butyrate metabolism (2), the pentose phosphate pathway (3), fructose and mannose metabolism (3), RNA degradation (4), pyruvate metabolism (3), and arginine and proline metabolism (2). The most significant pathways uniquely upregulated in JIA2 genes under moderate stress included gene replication (12), cyanide amino acid metabolism (13), pyrimidine metabolism (10), homologous recombination (5), nitrogen metabolism (6), and purine metabolism (8). The co-downregulated genes in BA9 and JIA2 under moderate stress were mainly associated with phenylpropane biosynthesis (39/95, with the numbers representing the genes in BA9 and JIA2, respectively); phenylalanine metabolism (17/29); biosynthesis of phenylalanine, tyrosine, and tryptophan (3/7); and cyanide amino acid metabolism (4/20). The most significant pathways uniquely downregulated in BA9 genes under moderate stress included the ribosome pathway (41), whereas, in JIA2, they involved starch and sucrose metabolism (50), plant–pathogen interactions (47), linoleic acid metabolism (10), plant hormone signal transduction (30), galactose metabolism (13), and flavonoid biosynthesis (11).

The metabolic pathways involved in the co-upregulation of BA9 and JIA2 genes under severe stress were mainly concentrated in carotenoid biosynthesis (10/5, with the numbers representing genes in BA9 and JIA2, respectively), plant hormone signal transduction (19/17), glycolysis (16/19), galactose metabolism (9/18), protein processing in the endoplasmic reticulum (20/28), linoleic acid metabolism (5/5), starch and sucrose metabolism (14/43), arginine and proline metabolism (6/11), butyrate metabolism (3/3), carbon fixation in photosynthetic organisms (5/10), and pyruvate metabolism (5/16). The most significant pathways uniquely upregulated in BA9 genes under severe stress included lipid metabolism (9), endocytosis (14), the spliceosome (13), pentose phosphate pathway (5), and β-alanine metabolism (4). In contrast, the pathways uniquely upregulated in JIA2 genes under severe stress included cyanide amino acid metabolism (18); DNA replication (11); homologous recombination (11); phenylpropane biosynthesis (33); fructose and mannose metabolism (10); alanine, aspartate, and glutamate metabolism (10); and RNA degradation (12). The metabolic pathways involved in the co-downregulation of BA9 and JIA2 genes under severe stress were mainly concentrated in phenylpropanoid biosynthesis (15/124), ubiquinone and other-terpenoid-quinone biosynthesis (2/16), alpha-linolenic acid metabolism (2/15), and tyrosine metabolism (3/10). The most significant pathways uniquely downregulated in BA9 genes under severe stress included the ribosome pathway (61) and the metabolism of cysteine and methionine (7). In contrast, pathways uniquely downregulated in JIA2 genes involved plant–pathogen interaction (83), phenylalanine metabolism (39), flavonoid biosynthesis (32), linoleic acid metabolism (14), plant hormone signal transduction (45), starch and sucrose metabolism (49), glutathione metabolism (29), circadian rhythm in plants (12), and biosynthesis of phenylalanine, tyrosine, and tryptophan (12).

### 2.6. Protein Functional Annotation Analysis

**Figure 8:**
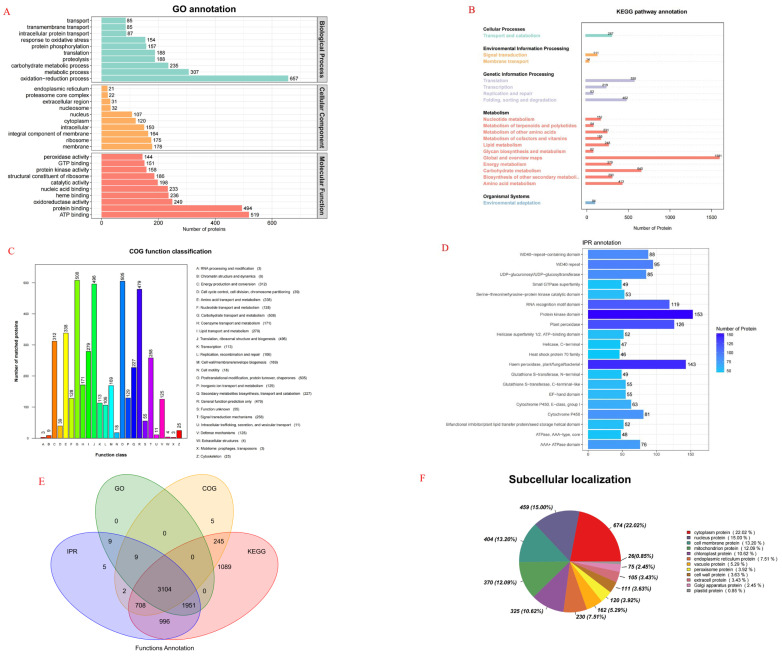
Function classification map of two oat cultivars with different drought tolerances under drought stress. Note: (**A**) is the GO function classification diagram; (**B**) is the KEGG classification diagram; (**C**) is the COG function classification diagram; (**D**) is the IPR function annotation; (**E**) is a petal diagram of each database’s classification; (**F**) is a subcellular location diagram.

Proteomic sequencing analysis was conducted on oat roots to identify key proteins and explore important pathways in oat seedlings with different drought resistances. After 0 and 7 days of moderate and severe stress, BA9 and JIA2 samples were collected for proteomic sequencing analysis. GO functional annotation was performed on the root tissues of two oat cultivars across all treatments (Figure 8A). A total of 5064 DEPs were annotated, involving 1104 entries. The top 10 categories are as follows: biological processes—oxidation–reduction, metabolic and carbohydrate metabolic processes, translation, proteolysis, protein phosphorylation, response to oxidative stress, intracellular protein transport, transport, and transmembrane transport; and cellular components—membranes, ribosomes, integral components of membranes, intracellular, cytoplasm, the nucleus, nucleosome, extracellular region, proteasome core complex, and the endoplasmic reticulum. The key molecular functions were oxidoreductase, catalytic, and peroxidase activities; ATP, GTP, RNA, protein, heme, and nucleic acid binding; and structural constant of the ribosome.

KEGG functional annotation was performed on oat root tissues from two cultivars across all treatments (Figure 8B), identifying a total of 8093 DEPs. These proteins were categorized into five hierarchical categories and 19 metabolic pathways: cellular processes—transport and catabolism; environmental information processing—signal transduction and membrane transport; genetic information processing—transcription, translation, replication, and repair, and folding, sorting, and degradation; metabolism—nucleotide, lipid, cofactors and vitamins, energy, carbohydrate, terpenoids and polyketides, other amino acids, global and overview maps, glycan biosynthesis, and secondary metabolites biosynthesis; and organic systems—environmental adaptation.

COG functional annotation was performed on oat root tissues from two cultivars across all treatments (Figure 8C), identifying 4073 DEPs. The annotation results (Figure 8C) revealed the following numbers of proteins in each category: carbohydrate transport and metabolism (508); post-translational modification, protein turnover, and chaperones (505); translation, ribosomal structure, and biogenesis (496); general function prediction only (479); amino acid transport and metabolism (338); energy production and conversion (312); lipid transport and metabolism (279); signal transduction mechanisms (258); secondary metabolite biosynthesis, transport, and catabolism (227); coenzyme transport and metabolism (171); cell wall, membrane, and envelope biogenesis (169); inorganic ion transport and metabolism (129); nucleic acid transport and metabolism (128); defense mechanisms (125); translation and transcription (113); replication, recombination, and repair (106); cell cycle control, cell division, and chromosome partitioning (39); cytoskeleton (25); cell motility (18); intracellular transport, secretion, and vesicle transport and circular traffic, secrecy, and vascular transport (11); chromatin structure and dynamics (9); extracellular structures (4); RNA processing and modification (3); and mobile groups—prophages and transposons (3) and function unknown (55).

IPR functional annotation was performed for oat root tissues from two cultivars across all treatments (Figure 8D), identifying 6784 DEPs. The annotation results indicated the following numbers of proteins in each category: protein kinase domain (153), heme peroxidase [plant/fungal/bacterial (143)], plant peroxidase (126), RNA recognition motif domain (119), WD40 repeat (95), domains containing WD40 repeat sequences and WD40 peak-containing domains (88), UDP-glucuronosyl/UDP-glucosyltransferase (85), cytochrome P450 (81), AAA + ATPase domain (76), glutathione-S-transferase [C-terminal like (55)], EF-hand domain (55), serine-threonine/tyrosine kinase catalytic domain (55), helicase superfamily 1/2 ATP binding domain (52), bifunctional inhibitor/plant lipid transfer protein/seed storage helical domain (52), small GTPase superfamily (49), glutathione S-transferase [N-terminal (49)], helicase C-terminal (47), heat shock protein 70 family (46), and ABC transporter-like (43).

The results of this study (Figure 8F) revealed 3061 DEPs, categorized into 12 categories: cytoplasmic proteins (674, 20.02%), nuclear proteins (459, 15.00%), cell membrane proteins (404, 13.20%), mitochondrial proteins (370, 12.09%), chloroplast proteins (325, 10.62%), endoplasmic reticulum proteins (230, 7.51%), vacuolar proteins (162, 5.29%), peroxisome protein (120, 3.92%), and others.

### 2.7. Identification of DEPs

**Figure 9:**
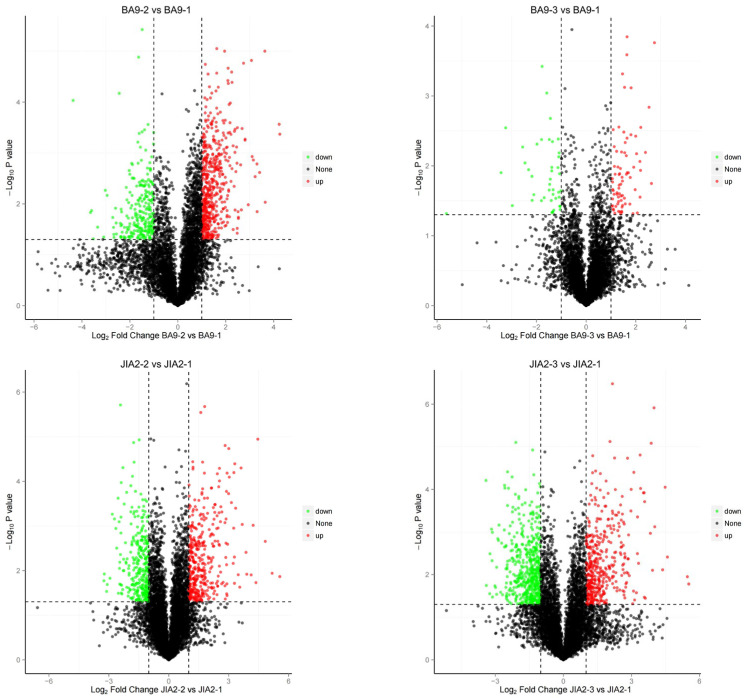
Volcano map of differentially expressed proteins of two cultivars under drought stress. Note: When FC ≥ 2.0 and *p*-value ≤ 0.05, we screened for upregulated-expression proteins, when FC ≤ 0.50 and *p*-value ≤ 0.05, we screened for downregulated-expression proteins. Note: BA9-1, BA9-2, BA9-3, JIA2-1, JIA2-2, and JIA2-3 represent the normal water supply (70%), moderate drought stress (45%), and severe drought stress (30%) conditions for BA9 and JIA 2, respectively.

**Figure 10:**
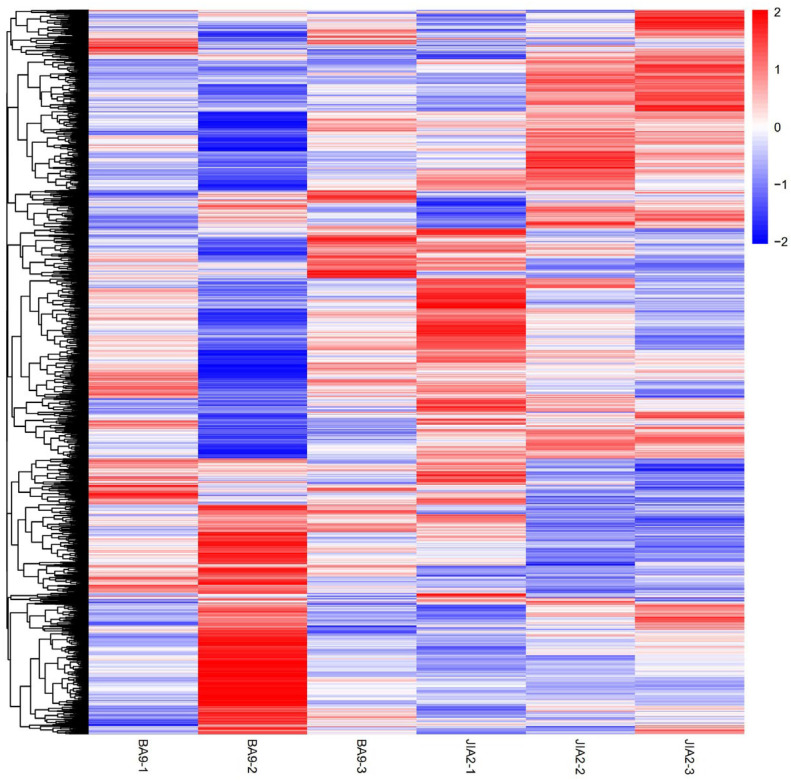
Cluster map of differentially expressed proteins of two cultivars under drought stress. Note: BA9-1, BA9-2, BA9-3, JIA2-1, JIA2-2, and JIA2-3 represent the normal water supply (70%), moderate drought stress (45%), and severe drought stress (30%) conditions for BA9 and JIA 2, respectively.

As depicted in Figure 9, under moderate stress, BA9 exhibited 590 upregulated and 397 downregulated DEPs, while under severe stress, BA9 exhibited 126 upregulated and 75 downregulated proteins. Under moderate stress, JIA2 exhibited 489 upregulated and 394 downregulated DEPs, while under severe stress, JIA2 exhibited 493 upregulated and 701 downregulated proteins. Significant differences were observed in the number of upregulated and downregulated proteins between BA9 and JIA2 under severe stress (Figure 10).

The DEPs co-expressed by BA9 and JIA2 under drought stress are listed in Table S3, with most displaying consistent expression trends between the two cultivars. Under drought stress, 314 specific DEPs were detected in JIA2. The numbers of upregulated and downregulated proteins were roughly equal, with functions primarily involving the structural constants of the ribosome, protein binding, oxidation–reduction processes, metabolic processes, integral membrane components, catalytic activity, carbohydrate metabolic processes, and other functions.

### 2.8. GO and KEGG Enrichment Analyses of DEPs

**Figure 11:**
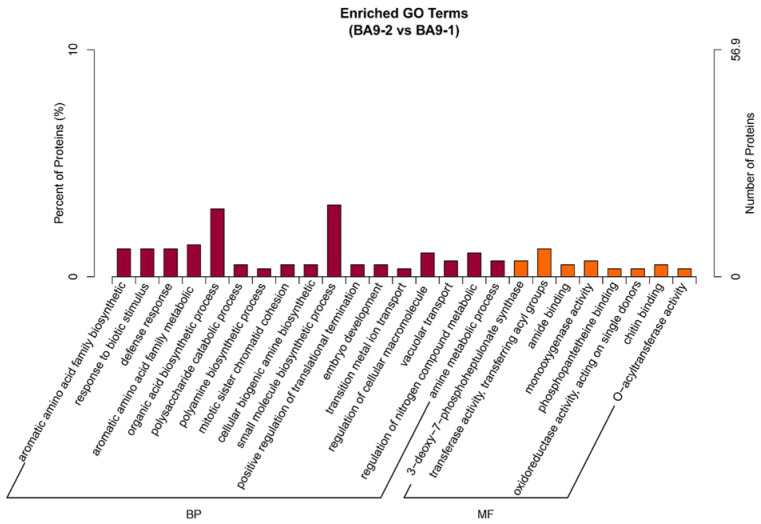

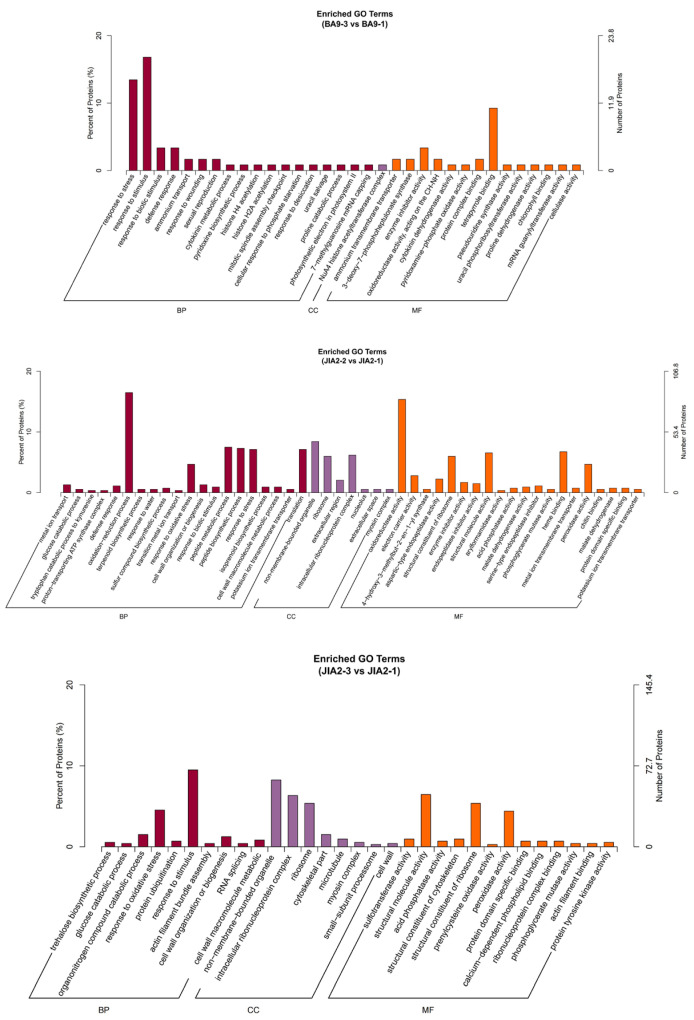
Analysis of GO enrichment of differentially expressed proteins in oats under drought stress. Note: BA9-1, BA9-2, BA9-3, JIA2-1, JIA2-2, and JIA2-3 represent the normal water supply (70%), moderate drought stress (45%), and severe drought stress (30%) conditions for BA9 and JIA 2, respectively.

GO classification of the DEPs indicated that under moderate stress, BA9 proteins were primarily enriched in the organic acid biosynthetic process, the small-molecule biosynthesis process, defense responses, regulation of the cellular macromolecular biosynthesis process, and transferase activity involving acyl groups other than aminoacyl groups. Under severe stress, BA9 proteins were predominantly enriched in response to stimuli, response to stress, tetrapyrrole binding, enzyme inhibitor activity, and defense response. Under moderate stress, JIA2 DEPs were mainly enriched in the oxidation–reduction process, oxidoreductase activity, intracellular nonmembrane-bound organelles, peptide metabolic processes, and response to stress. Under severe stress, JIA2 proteins were predominantly enriched in response to stimuli, intracellular nonmembrane-bounded organelles, structural molecular activity, structural constants of ribosomes, ribosomes, and peroxidase activity. These findings indicate that both oat cultivars experienced heightened biological, metabolic, and molecular functional responses under drought stress, with JIA2 undergoing more pronounced changes in cellular components compared to BA9 (Figure 11).

**Figure 12:**
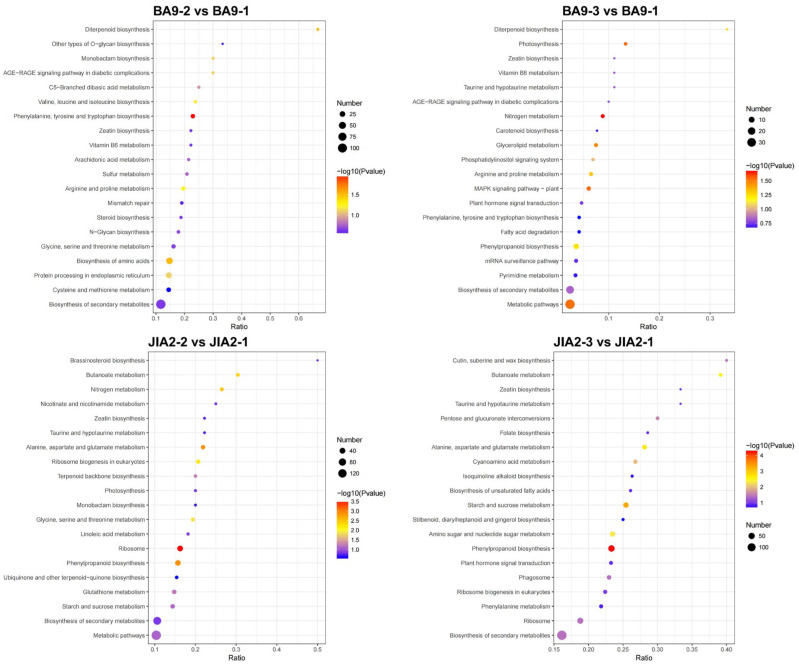
Analysis of KEGG enrichment of differentially expressed proteins in oats under drought stress. Note: BA9-1, BA9-2, BA9-3, JIA2-1, JIA2-2, and JIA2-3 represent the normal water supply (70%), moderate drought stress (45%), and severe drought stress (30%) conditions for BA9 and JIA 2, respectively.

We used the KEGG database to identify the main metabolic pathways associated with DEPs (Figure 12). Under moderate stress, the metabolic pathways associated with BA9 differential proteins mainly included the biosynthesis of secondary metabolites, amino acids, protein processing in the endoplasmic reticulum, and cysteine and methionine metabolism. Under severe stress, the metabolic pathways for BA9 differential proteins were primarily related to metabolic pathways, the biosynthesis of secondary metabolites, phenylpropanoid biosynthesis, the MAPK signaling pathway in plants, and nitrogen metabolism. For JIA2 differential proteins, moderate stress primarily involved metabolic pathways: the biosynthesis of secondary metabolites, ribosomes, phenylpropanoid biosynthesis, and amino sugar and nucleotide sugar metabolism. Under severe stress, the metabolic pathways for JIA2 differential proteins were mainly associated with the biosynthesis of secondary metabolites, ribosomes, phenylpropanoid biosynthesis, starch and sucrose metabolism, amino sugar and nucleotide sugar metabolism, and alanine, aspartate, and glutamate metabolism.

### 2.9. Transcriptomic and Proteomic Correlation Analysis of Oat Response to Drought Stress

**Figure 13:**
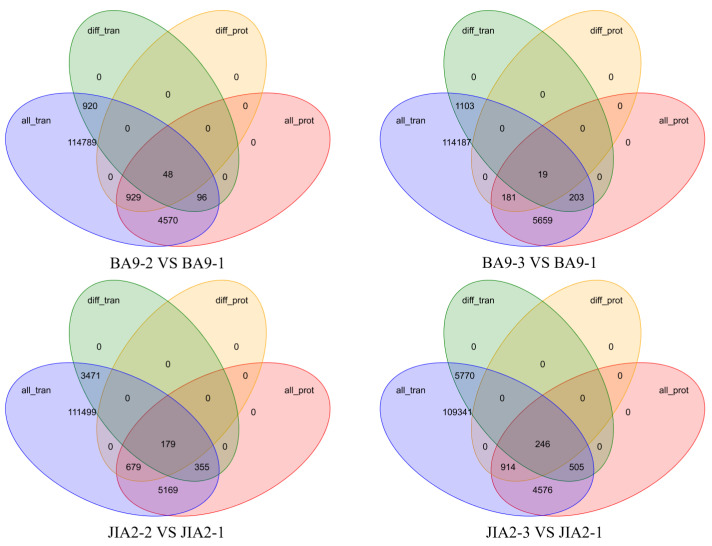
Differentially expressed genes and protein association analysis of two oat cultivars under drought stress. Note: BA9-1, BA9-2, BA9-3, JIA2-1, JIA2-2, and JIA2-3 represent the normal water supply (70%), moderate drought stress (45%), and severe drought stress (30%) conditions for BA9 and JIA 2, respectively.

Figure 13 presents the results of a correlation analysis concerning transcriptomics and proteomics. Compared with no drought stress, BA9 under moderate stress exhibited 1064 DEGs and 977 DEPs, with 48 matched cor DEGs-DEPs. Under severe stress, BA9 displayed 1325 DEGs and 200 DEPs, with 19 matched cor DEGs-DEPs. For JIA2, under moderate stress, 4005 DEGs and 858 DEPs were identified, among which 179 were matched with cor DEGs-DEPs. Under severe stress, JIA2 presented 6521 DEGs and 1160 DEPs, with 246 matched cor DEGs-DEPs.

**Figure 14:**
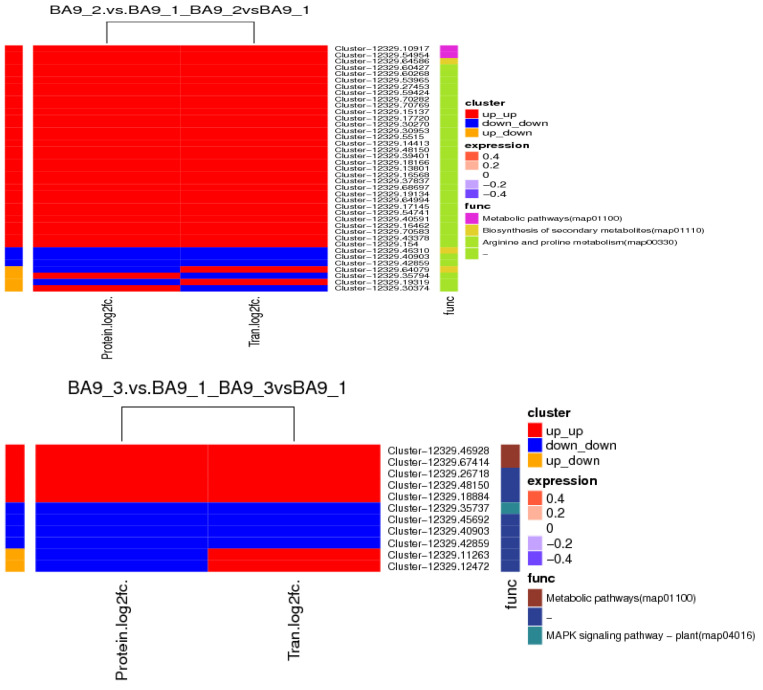
KEGG annotation of BA9 differentially expressed genes and protein association analysis under drought stress. Note: BA9-1, BA9-2, and BA9-3 represent the normal water supply (70%), moderate drought stress (45%) and severe drought stress (30%) conditions for BA9.

The KEGG functional enrichment results of the analysis of the association between DEGs and DEPs indicated that, compared with no drought stress, most DEGs and DEPs in BA9 were upregulated under moderate stress. Some DEGs and DEPs were downregulated, while a few exhibited opposing expression trends. The main pathways involved included metabolism (Cluster-12329.10917: V-type proton ATPase subunit; Cluster-12329.54954: assuming protein BRADI_1g03920v3), biosynthesis of secondary metabolites (Cluster-12329.64079: 6-phosphofructokinase 2; Cluster-12329.46310: Cytochrome P450 84A1; Cluster-12329.54450: peroxidase), and arginine and proline metabolism (Cluster-12329.60427: assuming protein BRADI_2g54920v3). Under severe stress, the number of DEGs and DEPs associated with the upregulation and downregulation of BA9 was roughly equal, with a relatively smaller total number. These primarily involved metabolic pathways (Cluster-12329.46928: β-glucosidase 4; Cluster-12329.60427: assuming protein BRADI_2g54920v3) and plant signal transduction pathways (Cluster-12329.35737: Abscisic acid receptor PYL2), as depicted in Figure 14.

**Figure 15:**
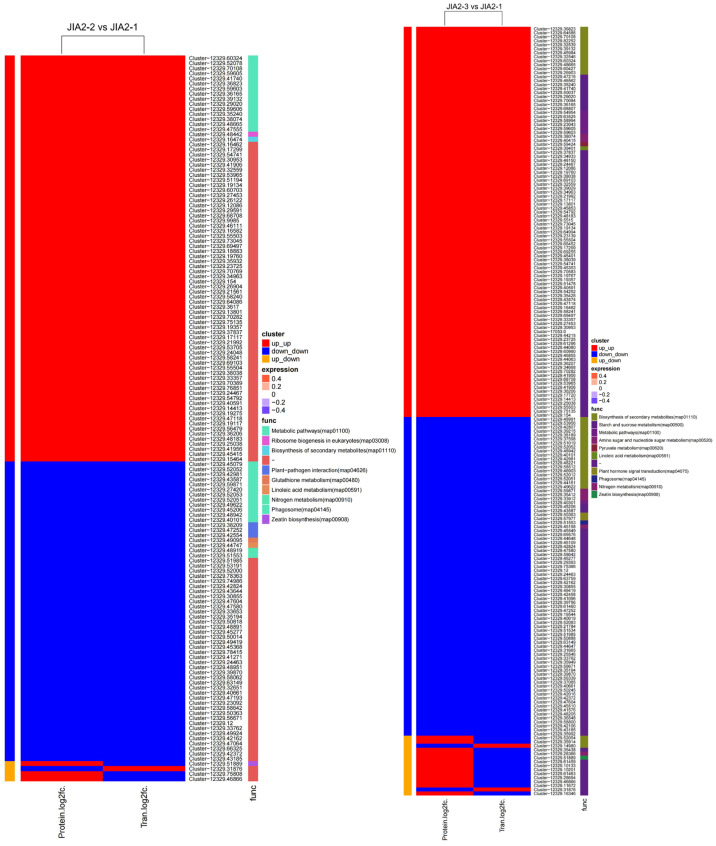
JIA2 differentially expressed genes and protein association analysis under drought stress KEGG annotation. Note: JIA2-1, JIA2-2, and JIA2-3 represent the normal water supply (70%), moderate drought stress (45%), and severe drought stress (30%) conditions for JIA 2.

Compared with no drought stress, under moderate stress, JIA2 exhibited 80 matched upregulated DEGs and DEPs, primarily associated with metabolic pathways, including Cluster-12329.52078 (peroxidase 7), Cluster-12329.10917 (V-type proton ATPase subunit), Cluster-12329.27420 (peroxidase P7), Cluster-12329.59603 (NADP-dependent malate enzyme), Cluster-12329.41740 (1,4-alpha-glucan branching enzyme 2), Cluster-12329.45206 (potential alpha trehalose phosphate synthase), Cluster-12329.36823 (blunt leaf alcohol 14-α-demethylase), Cluster-12329.35358 (3-phosphoglycerol 2-O-acyltransferase 6), and Cluster-12329.43587 (fructan exonuclease). Additional pathways included biosynthesis of secondary metabolites (Cluster-12329.16474: NAD(P)H dehydrogenase FQR1) and ribosome biogenesis in eukaryotes (Cluster-12329.48442). Furthermore, 59 matched DEGs and DEPs were downregulated, mainly involving metabolic pathways such as glutathione metabolism (Cluster-12329.49095: protein IN2-1), plant–pathogen interactions (Cluster-12329.47252: calcium-dependent protein kinase 13; Cluster-12329.42554: Predictive protein), linoleic acid metabolism (Cluster-12329.44747: lipoxygenase), nitrogen metabolism (Cluster-12329.48919), and the phagosome (Cluster-12329.51553). Additionally, a few matched DEGs displayed protein expression trends in the opposite direction (Figure 15).

Under severe stress, JIA2 exhibited 98 matched upregulated DEGs and DEPs, mainly associated with metabolic pathways, including Cluster-12329.59605 (NADP-dependent malate enzyme), Cluster-12329.58994 (hypothesized aldehyde dehydrogenase BIS1), Cluster-12329.63525 (mitochondrial succinate semialdehyde dehydrogenase), and Cluster-12329.59603 (NAD-dependent malate enzyme). Key pathways included starch and sucrose metabolism (Cluster-12329.47216: alpha glucan phosphorylase, H isoenzymes; Cluster-12329.35240: β-glucosidase 30; Cluster-12329.35438: hexokinase-7; Cluster-12329.68807: starch synthase 1; Cluster-12329.41740: 1,4-alpha glucan branching enzyme 2; Cluster-12329.99020: sucrose synthase 4), biosynthesis of secondary metabolites (Cluster-12329.36823: blunt-leaf alcohol 14-α-demethylase; Cluster-12329.35914: short-chain dehydrogenase/reductase 2b; Cluster-12329.82252: 3-phosphoglyceraldehyde dehydrogenase), linoleic acid metabolism (Cluster-12329.99020: sucrose synthase 4), pyruvate metabolism (Cluster-12329.9902), lactase glutathione lyase (Cluster-12329.59424), and amino and nucleotide sugar metabolism (Cluster-12329.38074: hexamine-A; Cluster-12329.40415: 26 kDa endonuclease 1). Additionally, 80 matched DEGs and DEPs were downregulated, primarily linked to metabolic pathways such as Cluster-12329.40301 (sorbitol dehydrogenase isoform X1), starch and sucrose metabolism (Cluster-12329.45206: potential α-trehalose phosphate synthase; Cluster-12329.43587: fructan exonuclease), biosynthesis of secondary metabolites (Cluster-12329.48221: cytochrome P450; Cluster-12329.40101: cationic peroxidase SPC4; Cluster-12329.58512: ortho aminobenzoate synthase α2 subunit; Cluster-12329.42981: peroxidase 50; Cluster-12329.44161: DSL esterase/lipase At5g45910), phagosomes (Cluster-12329.51553), plant hormone signal transduction (Cluster-12329.57977: potential serine/threonine protein kinase At4g35230; Cluster-12329.50363: predicted protein), amino sugar and nucleotide sugar metabolism (Cluster-12329.33912: α-L-arabinofuranosidase 1; Cluster-12329.59871: hypothesized protein BRADI_2g55620v3), and nitrogen metabolism (Cluster-12329.45198: Nitrate transporter protein). A small number of matched DEGs also exhibited opposite protein expression trends.

**Figure 16:**
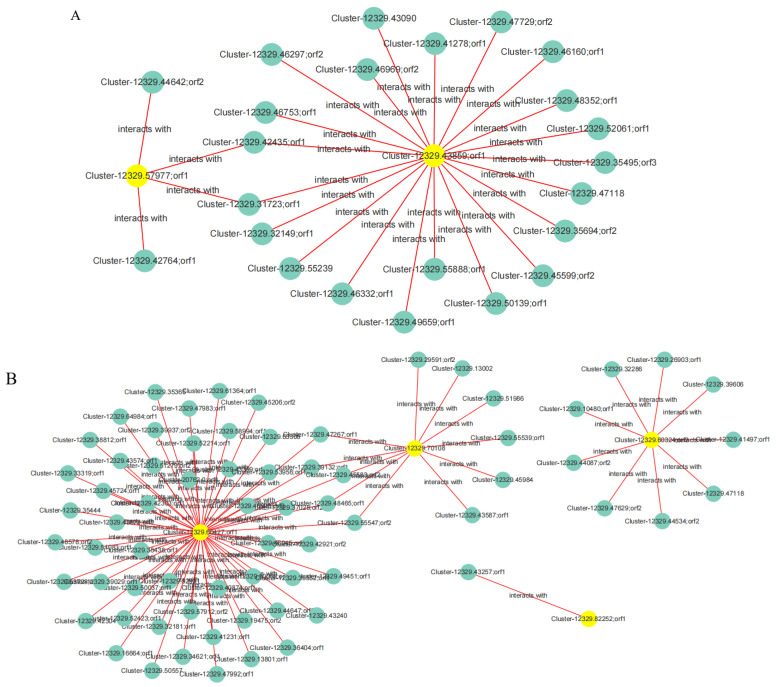

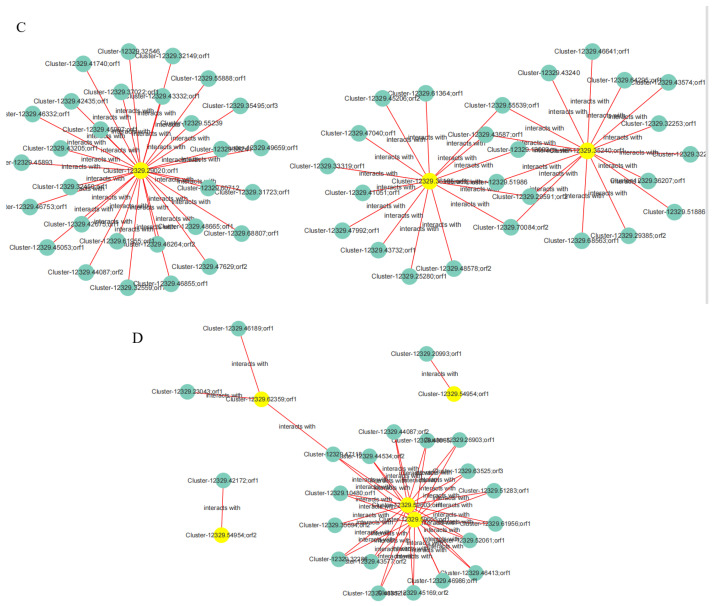
Mapping of key cor-DEG-DEP genes and protein networks. Note: (**A**–**D**) represent cor-DEGs-DEPs, which correspond to plant hormone signal transduction, secondary metabolite biosynthesis, carbohydrate metabolism process, and metabolic pathway function, respectively.

Based on the correlation between DEGs and DEPs, four types of interacting DEPs were identified in the gene–protein interaction network analysis of the drought-resistant cultivar JIA2 under severe stress compared with no drought stress. These proteins are mainly involved in plant hormone signal transduction, biosynthesis of secondary metabolites, carbohydrate metabolism, and metabolic pathways. Specific examples include the following (Figure 16).

(1) Cluster-12329.57977, Orf1 (serine/threonine protein kinase), associated with Cluster-12329.44642; Orf2, Cluster-12329.42764; Orf1, Cluster-12329.42435; Orf1, and Cluster-12329.31723. These proteins are involved in functions such as protein kinase activity. Cluster-12329.43859; Orf1 (histidine kinase), linked to Cluster-12329.46160; Orf1, Cluster-12329.49659; Orf1, Cluster-12329.46332; Orf1, Cluster-12329.46969; Orf2, Cluster-12329.35694; Orf2, Cluster-12329.47729; Orf2, Cluster-12329.48352; and Orf1, Cluster-12329.55239. These proteins are primarily involved in functions such as protein kinase activity, catalytic activity, glutamine synthetase, oxidoreductase activity, ATP binding, DNA binding, transcription, and thioredoxin.

(2) Cluster-12329.60427, Orf1 (glutamate 5-kinase) plays a key role and is associated with Cluster-12329.46488; Orf1 (hexokinase-2), Cluster-12329.52423; Orf1, Cluster-12329.55547; Orf2 (NADP-dependent malate enzyme), and Cluster-12329.47983; 51 proteins, including Orf1 (aspartate and glutamate aminotransferase), Cluster-12329.35444, and Cluster-12329.63308. These proteins are involved in functions including transmembrane transport, redox processes, amino acid transport and metabolism, and copper and metal ion binding. Cluster-12329.70108 (pyruvate decarboxylase) is associated with Cluster-12329.39132; Orf1 (ketotransferase), Cluster-12329.48465; Orf1 (aspartate aminotransferase), Cluster-12329.47267; Orf1 (pyruvate decarboxylase), Cluster-12329.55539; Orf1 (soluble acid-converting enzyme), and Cluster-12329.99591, with 20 proteins, including Orf2 (fructosyltransferase). These are primarily involved in carbohydrate transport and metabolism, amino acid transport and metabolism, energy production and conversion, and sugarcane candy glycosyltransferase. Cluster-12329.82252; Orf1 (phosphoglyceraldehyde dehydrogenase), was associated with Cluster-12329.43257, Orf1 (eukaryotic translation initiation factor).

(3) Cluster-12329.29020, Orf1 (sucrose synthase 4), plays a key role and is associated with Cluster-12329.42345, Orf1 (serine/threonine protein kinase SAPK8 isoform X1), Cluster-12329.46332; Orf1 (serine/threonine protein kinase STY13), Cluster-12329.45053; Orf1 (UDP pyrophosphate synthase), Cluster-12329.68807; Orf1 (starch synthase 1), and Cluster-12329.32559; Orf1 (α-glucosidase), among 39 proteins. These are primarily associated with protein kinase activity, carbohydrate transport and metabolism, and starch and sucrose metabolism. Cluster-12329.35240; Orf1 (β-glucosidase) is associated with Cluster-12329.43809; Orf2 (aminopeptidase M1-B), Cluster-12329.43240, Cluster-12329.32231; Orf1 (aldose 1-isomerase), Cluster-12329.68563; Orf1, Cluster-12329.36207; Orf1 (anthocyanin 3-O-glucosyltransferase), Cluster-12329.32253; Orf1 (Xet2 protein), numbering among 16 proteins. These are mainly involved in protein hydrolysis, heat shock proteins, glycolysis, carbohydrate transport and metabolism, flavonoid glycosyltransferases, and xylose glycosyltransferases. Cluster-12329.36165; Orf1 (1-phosphate glucosyltransferase) is associated with Cluster-12329.25280; Orf1, Cluster-12329.48578; Orf2 (glycerol kinase), Cluster-12329.43732; Orf1 (6-phosphogluconate lactonase), Cluster-12329.41051; Orf1 (cinnamoyl CoA reductase), Cluster-12329.47992; Orf1 (trehalose-6-phosphate synthase 6), among 16 proteins. The functions are primarily associated with oxidoreductase activity, energy production and conversion, carbohydrate metabolism, catalytic activity, and the trehalose biosynthesis process.

(4) Cluster-12329.62359; Orf1 (DNA primer subunit) was associated with Cluster-12329.23043; Orf1, Cluster-12329.46189; Orf1 and Cluster-12329.47118 (DNA Topoisomerase II), forming three proteins primarily involved in purine metabolism, chromosome separation ATPase, replication, recombination, and repair. Cluster-12329.59605; Orf1 (NADP-dependent malate enzyme) was associated with Cluster-12329.51283; Orf1, Cluster-12329.61956; Orf1, Cluster-12329.45169; Orf2 (protein RCF3 containing RNA binding KH domain), Cluster-12329.44534; Orf2 and 17 other proteins. These proteins are mainly associated with signal transduction mechanisms, energy production and conversion, nucleic acid binding, and redox processes. Cluster-12329.54954; Orf1 and Cluster-12329.54954; Orf2 are associated with Cluster-12329.20993; Orf1 (phosphoglycolic acid phosphatase 2) and Cluster-12329.42172; Orf1, primarily involving nucleotide transport and metabolism.

### 2.10. Validation of Key cor-DEGs-DEPs via qRT-PCR

**Figure 17:**
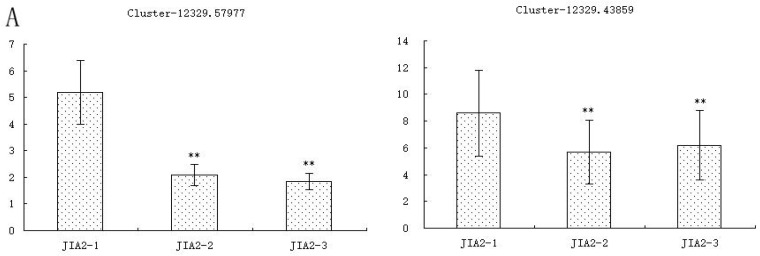

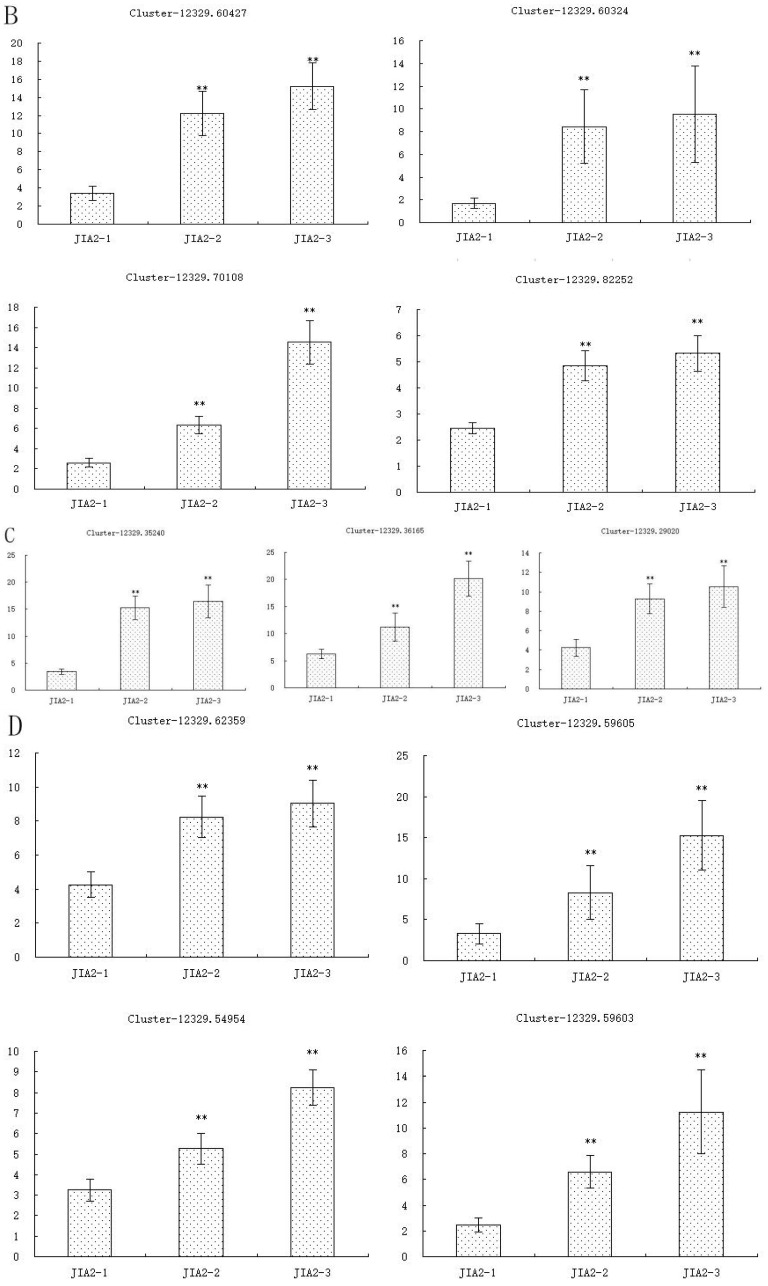
QRT-PCR expression of 13 target genes. Note: ** denotes significant differences (*p* ≤ 0.01) between the normal water supply and drought stress treatments. (**A**–**D**) represent cor-DEG-DEPs, which correspond to plant hormone signal transduction, secondary metabolite biosynthesis, carbohydrate metabolism process, and metabolic pathway function, respectively.

To analyze the expression patterns of related genes, 13 key cor DEGs-DEPs from four key pathways in the drought-resistant cultivar JIA2 were selected for qRT-PCR analysis (Figure 17). Two genes involved in plant hormone signaling transduction were downregulated. Two genes related to the biosynthesis of secondary metabolites and three genes associated with carbohydrate metabolism processes were upregulated. Additionally, four genes related to metabolic pathways were upregulated. The expression trends of these genes aligned with those of their corresponding proteins.

## 3. Discussion

### 3.1. Correlation Analysis Revealed Key Metabolic Pathways of the Oat in Response to Drought Stress

**Figure 18:**
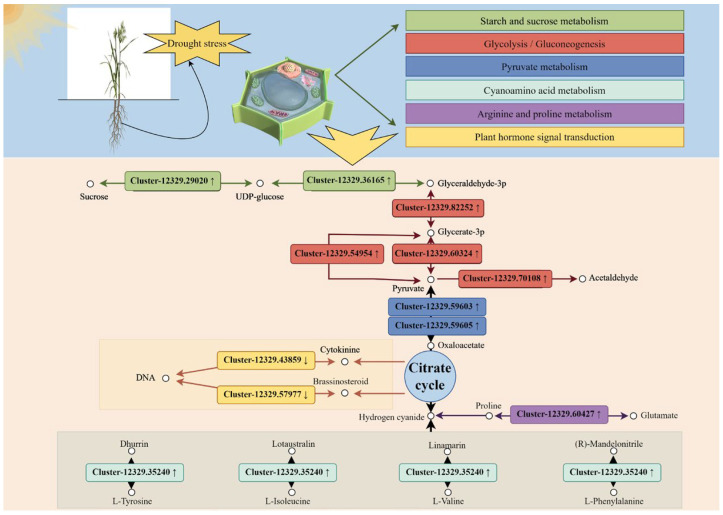
Schematic diagram of key metabolism response proteins and genes of drought-tolerant cultivar JIA2 in response to drought stress. Note: The colors of the genes at the bottom of the figure correspond to the metabolic pathways of the same color at the top (this figure was created using Figdraw).

Proteomics is widely utilized to comprehensively examine protein changes under stress, uncover their mechanisms of action, and identify potential biomarkers. However, the number of related studies remains limited [21], potentially due to RNA-seq detection being more sensitive than protein detection or due to factors such as post-transcriptional and post-translational modifications or protein-regulated degradation [22]. To further investigate the regulatory mechanisms of the two oat cultivars under drought stress, we interpreted the effects of drought stress through an in-depth combined analysis of the transcriptome and proteome under drought stress conditions. This approach offers the advantage of establishing robust connections between datasets by comparing and reusing the same samples across multiple omics and biological replicates. The findings indicate that drought stress significantly alters the metabolic pathways in oat roots, including the biosynthesis of secondary metabolites, plant hormone signal transduction, and carbohydrate metabolism processes (Figure 18). Correlation analysis of transcriptomics and proteomics revealed significant molecular-level changes in the drought-resistant cultivar JIA2 and the sensitive cultivar BA9, aligning with previous findings on their growth responses under drought stress.

### 3.2. Carbohydrate Metabolism

Carbohydrate metabolism encompasses numerous biochemical processes essential for the synthesis, decomposition, and interconversion of carbohydrates in organisms, significantly influencing plant growth and stress responses [23]. The DEGs and DEPs identified in this study are involved in various forms of carbohydrate metabolism, with the highest number being associated with “starch and sucrose metabolism”. The carbon assimilation process primarily facilitates the synthesis of osmoregulatory substances, while the degradation of starch into glucose contributes to osmoregulation, enabling plants to resist or adapt to drought stress [24]. In the drought-resistant cultivar JIA2, the expression levels of soluble-sugar-metabolism-related genes exhibited significant changes, with upregulation and downregulation, suggesting that drought stress promotes starch degradation and soluble sugar accumulation, redirects carbon flow within cells, and supplies energy for drought resistance and adaptation. The results from association analysis and protein–gene network mapping further highlighted the critical roles of proteins such as Cluster-12329.29020; Orf1 (sucrose synthase 4), Cluster-12329.68807; Orf1 (starch synthase 1), Cluster-12329.32559; Orf1 (α-glucosidase) and Cluster-12329.36165; Orf1 (1-phosphate glucosyltransferase) in the adaptation of the oat to drought stress. Similar findings were reported in studies examining the expression changes of key enzyme genes involved in insoluble and soluble sugar metabolism in tea plants under drought stress [24].

### 3.3. Amino Acid Metabolism and Secondary Metabolism

Studies have identified that DEGs in Populus euphratica specimens subjected to drought stress are associated with amino acid metabolism and transport, with plasma membrane transporters facilitating amino acid transport [24]. In this experiment, the correlation analysis indicated that the biosynthesis of metabolites, amino sugar, and nucleotide sugar metabolism pathways were significantly enriched only in the drought-resistant cultivar JIA2. This indicates that these metabolic pathways play an important role in the drought resistance of JIA2. Furthermore, most of the DEGs and DEPs involved in glutamate and aspartate metabolism in JIA2, such as Cluster-12329.60427 (glutamate 5-kinase) and Cluster-12329.47983 (aspartate and glutamate aminotransferase), exhibited both upregulation and downregulation under drought stress. These findings suggest that glutamate and aspartate metabolism may be key regulatory pathways in the response of the common oat to drought stress.

Drought stress exerts a significant impact on the synthesis of secondary metabolites. DEGs associated with the metabolic pathways of various secondary metabolites, particularly key regulatory genes in the biosynthesis pathways of flavonoids and phenylpropanoids, were identified in the transcriptome of tea plants under drought stress [25]. The findings of this experiment align with those of previous studies. In oat roots, drought stress significantly affects the phenylpropanoid biosynthesis pathway, with most genes, including those corresponding to 4-coumaric acid CoA ligase, 3-hydroxyisobutyrylCoA hydrolase, caffeoyl CoA O-methyltransferase, peroxidase family proteins, and cationic peroxidase, exhibiting significantly higher downregulation than upregulation. Under drought stress, plants adapt metabolically through complex recombination across multiple metabolic pathways. Cytoplasmic enzyme activity related to defense and secondary metabolism in the root system undergoes significant changes under abiotic stress [26,27]. This experiment identified several proteins, including chitinase, 3-hydroxyisobutyryl-CoA hydrolase, 1-pyrroline-5-carboxylic acid dehydrogenase, 4-coumaric acid CoA ligase, keto transferase, and sulfotransferase, as being significantly differentially expressed only in the drought-resistant cultivar JIA2. These stress-induced proteins play important roles in secondary metabolism and key stress adaptation mechanisms, enhancing the drought resistance of the oat.

### 3.4. Plant Hormone Signaling and Transcription Factors

Plant hormones are closely linked to various abiotic stresses and play an important role in rehydration after drought stress [12,28]. Drought stress induced the expression of most genes involved in ABA biosynthesis in the root systems of the tested oat cultivars, including sucrose non-fermenting 1-related kinase, abscisic acid receptor, serine/threonine kinase SAPK1 protein, and abscisic acid receptor. Additionally, protein phosphatase 2C protein exhibited both upregulation and downregulation, while ABA-responsive element binding factors were downregulated. These findings indicate that ABA is likely a key regulatory factor in the response of the oat to drought stress [7,29]. Furthermore, the abscisic acid receptor PYL2 was specifically expressed in the sensitive cultivar BA9, whereas BR signaling kinase was significantly differentially expressed only in the drought-resistant cultivar JIA2.

AP2/ERF, MYB, NAC, bHLH, and WRKY transcription factors in the TF family play important roles in regulating abiotic stress tolerance in Arabidopsis and rice [30]. In this study, we observed no DEGs in BA9 under drought stress when compared with the Arabidopsis transcription factor database. Under moderate stress, JIA2 exhibited 3 upregulated and 9 downregulated DEGs in comparison to the database. These transcription factors were divided into 10 categories, mainly distributed as follows: MYB (2), WRKY (2), AP2 (1), GATA (1), and HSF (1). Under severe stress, JIA2 exhibited 5 upregulated and 12 downregulated DEGs, grouped into 12 categories, predominantly WRKY (3), MYB (2), HSF (2), bHLH (2), and GATA (1) [23]. In Populus euphratica, 19 TF families were identified, predominantly AP2/ERF and WRKY, followed by bZIP, NAC, MYB, bHLH, C2H2, and HSF families. In Vernicia lanceolata, MYB, ERF, and NAC significantly contribute to abiotic stress resistance [31]. Under drought stress, JIA2 transcription factors demonstrated both downregulation and upregulation, with downregulated factors such as MYB, WRKY, and HSF being more numerous. Various transcriptional regulatory mechanisms are involved in drought stress signal transduction pathways and stress responses in oats. Different genes within the same family exhibit diverse expression patterns under drought stress, reflecting their role in positive or negative regulation of stress response. Previous studies have also noted that a single transcription factor can interact with one or more members of the same or different families, indicating the complexity of transcription factors in plant drought stress response [32,33].

## 4. Materials and Methods

### 4.1. Experimental Materials

We experiment utilized the drought-resistant cultivar JiaYan 2 (JIA2) and water-sensitive cultivar BaYou 9 (BA9), both of which were preserved and bred in our laboratory.

### 4.2. Experimental Method

#### 4.2.1. Oat Drought Stress Experiment

The experiment was conducted in a greenhouse at the Oat Industry Research Center of Inner Mongolia Agricultural University using potted plants. The soil used in the pots was sandy loam with a field water-holding capacity of 16%. Oats were cultivated in plastic buckets measuring 25 cm in height, 24 cm in upper diameter, and 22 cm in lower diameter. Each bucket was filled with 10 kg (dry soil weight) of soil collected from the field tillage layer, and 3 g of diammonium phosphate and 3 g of urea were placed at the base. No topdressing was applied during the growth period. Before sowing, 1.5 L of water was added to moisten the bottom soil. Fifty seeds were sown per barrel, and 25 seedlings were maintained per barrel at the three-leaf stage. Two oat cultivars and three water stress gradients were set up, resulting in six treatments, and each treatment was repeated eight times. Drought stress was applied during the jointing stage for a total of 48 barrels. The buckets were completely randomized, with their positions changed weekly. The three water gradients were 30% field capacity (severe stress), 45% field capacity (moderate stress), and 70% field capacity (normal water supply). Treatments were designated as BA9-1, BA9-2, BA9-3, JIA2-1, JIA2-2, and JIA2-3, representing normal water supply (70%), moderate drought stress (45%), and severe drought stress (30%) conditions for BA9 and JIA2, respectively. Starting at the jointing stage (40 days after emergence), a 7-day water control treatment was applied daily using the weight difference method once each treatment reached the set water gradient. Fresh mixed root tissues extracted from total RNA and analyzed via proteomics were immediately frozen in liquid nitrogen and stored at −80 °C. We performed three replicate experiments for the RNA-seq and proteome analysis.

#### 4.2.2. Transcriptome Sequencing Analysis of Oat Root

##### Extraction and Qualification of RNA

Total RNA was isolated by treating samples with TRIzol (Invitrogen, Carlsbad, CA, USA). Complimentary DNA was synthesized from the RNA using the Reverse Transcriptase M-MLV Kit (TaKaRa, Beijing, China). The RNA was analyzed using a NanoPhotometer^®^ spectrophotometer (IMPLEN, Munich, Germany) and resolved on 1% agarose gels to verify shearing and quality. Library preparation and deep sequencing were conducted at Novogene Bioinformatics Technology Co., Ltd. (Beijing, China).

##### Transcriptome Sequencing and Quality Control

We used 1.5 µg of RNA per sample in the RNA sample preparation process. The NEBNext^®^ Ultra™ RNA Library Prep Kit for Illumina^®^ (NEB, Ipswich, MA, USA) was used to prepare sequencing libraries. Sequencing was performed using the Illumina Hiseq platform. Raw sequencing data were filtered using an integrated approach: we trimmed for reads with N constituents, adapter contamination, and low-quality reads with 50% of bases with Qphred ≤ 20.

##### Gene Annotation, Different Expression, and Enrichment Analyses

We assembled the trimmed reads using Trinity (version 2.5.1). The number of reads mapped to the reference genome was calculated using RSEM. Gene expression levels were estimated based on gene length and the number of reads expressed as fragments per kilobase of exon per million mapped reads (FPKM). Parallelism among replicate groups was assessed using Pearson correlation analysis. Differential gene expression analysis between the two conditions/groups was conducted using the DESeq2 package (1.38.3). *p*-values were adjusted using Benjamini and Hochberg’s approach to control the false discovery rate. Genes with an adjusted *p*-value < 0.05, as determined using DESeq2, were considered differentially expressed. Kyoto encyclopedia of genes and genomes (KEGG) and gene ontology (GO) analyses were subsequently performed to predict the pathways and functions of the DEGs.

##### qRT-PCR Analysis

All qRT-PCR experiments were conducted in triplicate using a LightCycler 480 instrument (Roche Applied Science, Mannheim, Germany). A total of 13 candidate DEGs involved in four key pathways were selected for qRT-PCR validation. Specific primer pairs for the selected genes were designed and are listed in Table S5. mRNA levels were calculated using the 2^–ΔΔCt^ method, and results are presented as mean ± standard error of the mean. Statistical analysis was performed using one-way ANOVA.

#### 4.2.3. Application of Label-Free Technology for Oat Root Proteome Sequencing

##### Extraction of Total Protein from Oat Roots

The frozen samples were crushed using a liquid-nitrogen-precooled crusher and ground further with liquid nitrogen. The resulting powder was mixed with lysis buffer in a 1:10 (*w*/*v*) ratio and vortexed. Ultrasonication was performed for 60 s, alternating 0.2 s on and 2 s off at 22% amplitude. Proteins were extracted at room temperature for 30 min, followed by centrifugation at 15,000× *g* for 1 h at 10 °C. The supernatant was collected, divided, and stored at −80 °C.

##### Protein Quantification

Protein concentrations were determined using the Bradford method. The concentrations (μg μL^–1^) were calculated based on the curve formula. For detailed procedural steps, please refer to the study.

Proteolysis (Filter-Aided Sample Preparation)

After protein quantification, 200 μg protein solution was transferred to a centrifuge tube, and DTT was added to obtain a final concentration of 25 mmol L^–1^. The solution was then reacted at 60 °C for 1 h, and then iodoacetamide was added to obtain a final concentration of 50 mmol L^–1^. The solution was maintained at room temperature for 10 min. After reductive alkylation, the protein solution was transferred to a 10 K ultrafiltration tube and centrifuged at 12,000× *g* for 20 min. The filtrate was collected, and 100 μL dissolution buffer was added. This step was repeated three times, and the filtrate was discarded after each centrifugation. Trypsin was then added to a new ultrafiltration tube, yielding a solution with a total protein mass of 4 μg (with a trypsin-to-protein ratio of 1:50) and volume of 50 μL. The reaction was incubated overnight at 37 °C. The following day, the solution was centrifuged at 12,000× *g* for 20 min, and the peptide solution in the filtrate was collected after enzymatic digestion. An additional 50 μL dissolution buffer was added to the ultrafiltration tube, and the tube was subjected to centrifugation at 12,000× *g* for 20 min. The resulting solution was combined with the previously collected peptide solution, yielding a total volume of 100 μL in the collection tube after enzymolysis. Finally, the solution was lyophilized in preparation for loading.

##### Nano-Upgraded Reversed-Phase Chromatography–Q Exactive Spectrometry for Protein Analysis

A solution with a volume of 20 μL containing 2% methanol and 0.1% formic acid was prepared for this experiment. The solution was centrifuged at 12,000× *g* for 10 min, and the supernatant was collected for sample loading. A sample volume of 10 μL was loaded using a loading pump with a flow rate of 350 nL min^–1^ for 15 min, and the separation flow rate was 300 nL min^–1^.

##### Mass-Spectrometry Data Analysis

The UniProt-Pooideae361804_20170619.fasta database (362,934 sequences) was used for analysis. Mass-spectrometry analysis was performed with a Thermo Q-Exactive mass spectrometer. Peptide Spectrum Matches with reliability exceeding 95% were considered trusted, while proteins containing at least one unique peptide were designated as trusted proteins. This study included only trusted peptides and proteins, with FDR verification applied to exclude peptides with FDR greater than 1% and egg whites. Proteins differing between paired samples across replicate groups were analyzed, and the mean value of the different multiples was used to quantify differences between samples. A t-test was conducted to calculate the *p*-value, which was used as the significance index.

##### Data Processing and Bioinformatics Analysis

Microsoft Excel 2010 and Statistical Analysis System (version 9.0) software were used for statistical analysis. Common functional database annotations for the identified proteins were conducted using COG, GO, and KEGG databases. Differential protein functional analyses, including GO and KEGG enrichment analyses, were performed for the selected differentially expressed proteins (DEPs).

## 5. Conclusions

Transcriptomic analysis revealed that under drought stress, JIA2 exhibited a higher number of DEGs compared with BA9. Most DEGs in JIA2 were downregulated, primarily associated with redox reactions, carbohydrate metabolism, plant growth hormone signaling regulation, and secondary metabolism. Proteomic analysis indicated significant differences in the number of upregulated and downregulated DEPs between the two cultivars under severe drought stress. A total of 314 specific DEPs were identified in JIA2, with an approximately equal number of upregulated and downregulated proteins. These proteins were predominantly involved in functions such as the maintenance of the structural constant of the ribosome, protein binding, the oxidation–reduction process, the maintenance of an integral component of membrane, catalytic activity, metabolic processes, and carbohydrate metabolic processes. The KEGG functional enrichment analysis of cor DEG-DEPs indicated that under moderate stress, most DEGs and proteins in BA9 were upregulated, while some were downregulated, with a few exhibiting opposing expression trends. These are mainly related to metabolic pathways, secondary metabolites biosynthesis, and pathways for arginine and proline metabolism. Under severe stress, BA9 demonstrated a roughly equal number of upregulated and downregulated DEGs and DEPs, with the total number being relatively small. These were primarily associated with metabolic pathways and plant signal transduction pathways. Under moderate stress, JIA2 upregulated cor DEGs-DEPs, primarily involving metabolic pathways, biosynthesis of secondary metabolites, and ribosome biogenesis in eukaryotes. Downregulation of cor DEGs and DEPs mainly involved metabolic pathways such as glutathione metabolism, plant–pathogen interactions, linoleic acid metabolism, nitrogen metabolism, and phagocytosis. Under severe stress, JIA2 upregulated cor DEGs and DEPs, mainly involving metabolic pathways, starch and sucrose metabolism, biosynthesis of secondary metabolites, linoleic acid metabolism, pyruvate metabolism, and amino sugar and nucleotide sugar metabolism pathways. Downregulation of cor DEGs and DEPs primarily involved metabolic pathways such as starch and sucrose metabolism, biosynthesis of secondary metabolites, phagosomes, plant hormone signal transduction, and nitrogen metabolism. Under severe stress, JIA2 exhibited four types of DEPs that interacted with each other in differential gene–protein interaction network analysis. These proteins are mainly involved in plant hormone signal transduction, the biosynthesis of secondary metabolites, carbohydrate metabolism processes, and metabolic pathways, including 13 key cor DEGs and DEPs. Future research should focus on further exploring DEGs and molecular markers related to these pathways, thereby contributing to the breeding of drought-resistant oat cultivars.

## Figures and Tables

**Table 1:** Differentially expressed transcription factors of JIA2 under moderate drought stress (JIA2-2).

Gene Id	JIA2-1 FPKM	JIA2-2 FPKM	log2FC	q-Value	Family
Cluster-12329.30654	114.83	21.46	−2.53	0.000014	WRKY
Cluster-12329.9983	3.06	0.94	−1.81	0.008212	WRKY
Cluster-12329.32221	14.72	7.21	−1.14	0.005371	TALE
Cluster-12329.64361	10.23	2.90	−1.93	0.010695	MYB_related
Cluster-12329.56093	33.69	4.58	−2.99	0.000007	MYB
Cluster-12329.75409	16.84	0.58	−4.98	0.011413	MYB
Cluster-12329.25874	59.96	20.78	−1.65	0.000001	LBD
Cluster-12329.79361	4.14	0.95	−2.23	0.028308	HSF
Cluster-12329.54718	3.48	10.69	1.50	0.000001	HD-ZIP
Cluster-12329.44206	6.38	18.83	1.44	0.008599	GATA
Cluster-12329.33174	17.00	8.22	−1.16	0.000132	Dof
Cluster-12329.34327	3.62	9.08	1.21	0.014410	AP2

**Table 2:** Differentially expressed transcription factors of JIA2 under severe drought stress (JIA2-3).

Gene Id	JIA2-1 FPKM	JIA2-3 FPKM	log2FC	q-Value	Family
Cluster-12329.30654	114.83	4.11	−4.94	0.000001	WRKY
Cluster-12329.9983	3.06	0.97	−1.78	0.008281	WRKY
Cluster-12329.53056	34.44	10.04	−1.91	0.033821	WRKY
Cluster-12329.71925	7.99	1.75	−2.32	0.000012	NAC
Cluster-12329.64361	10.23	2.23	−2.33	0.000501	MYB_related
Cluster-12329.56093	33.69	1.14	−5.02	0.000001	MYB
Cluster-12329.75409	16.84	0.20	−6.55	0.006993	MYB
Cluster-12329.25874	59.96	13.16	−2.32	0.000001	LBD
Cluster-12329.79361	4.14	0.65	−2.80	0.003270	HSF
Cluster-12329.40676	2.91	12.27	1.95	0.005442	HSF
Cluster-12329.54718	3.48	12.03	1.66	0.000203	HD-ZIP
Cluster-12329.44206	6.38	19.00	1.45	0.007739	GATA
Cluster-12329.33174	17.00	6.40	−1.54	0.000001	Dof
Cluster-12329.51794	1.08	5.33	2.16	0.006461	bHLH
Cluster-12329.49710	85.74	38.20	−1.30	0.024487	bHLH
Cluster-12329.54360	9.82	4.58	−1.23	0.008187	ARF
Cluster-12329.34327	3.62	9.86	1.31	0.000944	AP2

## Data Availability

Data are contained within the article and its Supplementary Materials. Proteomic and transcriptomic data can be found in the Oat Base database (http://112.126.28.109:8086, accessed on 15 January 2025).

## Supplementary Materials

The following supporting information can be downloaded at: https://www.mdpi.com/article/10.3390/plants14050792/s1, Table S1: The co-express differential genes under drought stress of BA9 and JIA2; Table S2: The specifically expresses of JIA2 differential genes under drought stress; Table S3: The co-express differential proteins of BA9 and JIA2 under drought stress; Table S4: The specifically expresses differential proteins of JIA2 under drought stress; Table S5: Primers used in real-time quantitative reverse transcription PCR (qRT-PCR) experiment.
