# Supplementary material for: Utilizing Multi-Omics Analysis to Elucidate the Molecular Mechanisms of Oat Responses to Drought Stress
Source: Plants (Basel). 2025 Mar 4;14(5):792. doi: 10.3390/plants14050792 (PMC11901947; doi:10.3390/plants14050792)
Supplement: Supplementary file 1 [file plants-14-00792-s001.zip › plants-3457518-supplementary.pdf]

Table S1. The co-express differential genes under drought stress of BA9 and JIA2.

Gene id	log2FC/BA9-1		log2FC/JIA2-1		Description
	BA9-2	BA9-3	JIA2-2	JIA2-3	
Cluster-12329.57294	2.9	2.0	1.8	1.8	26 kDa endochitinase 1-like
Cluster-19271.0	-6.9	-5.9	-4.6	-7.0	40S ribosomal protein S6-B-like
Cluster-12329.5335	-7.5	-7.4	-4.3	-6.8	60S ribosomal protein L3
Cluster-25393.0	-4.7	-5.6	-3.7	-7.6	60S ribosomal protein L8-1-like
Cluster-12329.64025	4.1	5.2	5.9	9.1	ABA-inducible protein PHV A1-like
Cluster-12329.18166	5.2	5.8	4.6	7.3	ABA-inducible protein PHV A1-like
Cluster-12329.29340	-3.4	-5.2	-2.8	-4.7	actin 1
Cluster-12329.38263	-2.2	-1.6	-1.7	-1.5	alcohol dehydrogenase 3-like
Cluster-12329.7678	-5.2	-3.4	-7.4	-6.4	alpha-humulene synthase-like
Cluster-12329.42859	-1.9	-2.1	-1.5	-2.4	ammonium transporter AMT2.1
Cluster-12329.47412	2.0	1.3	1.0	1.5	AT-hook motif nuclear-localized protein 27-like
Cluster-12329.52341	-1.6	-1.4	-2.0	-2.2	bidirectional sugar transporter SWEET4
Cluster-12329.58240	1.5	1.3	2.8	3.4	bifunctional epoxide hydrolase 2-like
Cluster-12329.39029	1.3	1.9	1.2	2.9	CSC1-like protein HYP1
Cluster-12329.14410	1.7	1.8	1.3	1.4	dehydrin
Cluster-12329.43993	2.0	2.9	2.7	4.7	dehydrin-/LEA group 2-like protein
Cluster-12329.71405	2.5	3.0	1.5	2.3	delta-1-pyrroline-5-carboxylate synthase-like
Cluster-12329.58767	-1.4	-1.4	-2.5	-3.9	disease resistance protein RPM1 isoform X1
Cluster-12329.43994	2.8	3.9	4.5	6.7	drought acclimation dehydrin WZY2, partial
Cluster-12329.29658	-3.4	-5.3	1.7	-3.4	ervatamin-B-like isoform X1
Cluster-12329.40430	-2.3	-2.7	-3.7	-4.9	ethylene response factor
Cluster-12329.27606	3.2	4.5	3.0	4.3	eukaryotic peptide chain release factor subunit 1-2-like
Cluster-12329.32433	1.6	1.1	1.7	2.6	expansin-A11 isoform X1
Cluster-12329.42428	-3.7	-4.4	-3.0	-6.2	extensin-like
Cluster-12329.59588	1.5	1.6	1.1	2.6	G-box-binding factor 3 isoform X1
Cluster-12329.7992	-1.6	-1.2	-2.7	-3.8	G-type lectin S-receptor-like serine/threonine-protein kinase At1g34300
Cluster-12329.69532	3.0	3.6	2.1	3.9	Heat stress transcription factor C-2b
Cluster-12329.45966	-2.0	-1.9	-2.6	-4.0	heavy metal-associated isoprenylated plant protein 43-like
Cluster-12329.72154	2.3	3.8	2.4	3.8	hypothetical protein
Cluster-12329.58613	6.3	5.7	3.4	5.0	hypothetical protein BRADI_1g09260v3
Cluster-12329.6269	-2.8	-3.4	-2.1	-2.8	hypothetical protein BRADI_1g10635v3
Cluster-12329.47668	-1.7	-1.8	-1.9	-2.0	hypothetical protein BRADI_1g31700v3
Cluster-12329.16385	-1.4	-1.0	-3.0	-3.5	hypothetical protein BRADI_1g46170v3
Cluster-12329.16224	3.0	2.2	2.3	3.1	hypothetical protein BRADI_1g65230v3
Cluster-12329.6655	-2.3	-3.3	-4.6	-4.1	hypothetical protein BRADI_2g00467v3
Cluster-12329.71455	3.0	3.2	2.8	4.4	hypothetical protein BRADI_2g17200v3
Cluster-12329.14312	3.6	5.0	3.4	4.7	hypothetical protein BRADI_2g27180v3
Cluster-12329.10582	2.3	3.4	3.2	4.8	hypothetical protein BRADI_2g27810v3
Cluster-12329.48609	-1.8	-1.9	-1.4	-2.8	hypothetical protein BRADI_2g52970v3
Cluster-12329.76811	-1.9	-2.3	-2.1	-3.6	hypothetical protein BRADI_2g54660v3
Cluster-12329.60427	2.1	2.4	1.8	2.6	hypothetical protein BRADI_2g54920v3
Cluster-12329.16260	2.7	3.4	2.5	4.6	hypothetical protein BRADI_3g22635v3
Cluster-12329.38859	-1.5	-2.8	-2.7	-3.9	hypothetical protein BRADI_4g02793v3
Cluster-12329.44891	-1.3	-1.3	-1.5	-2.3	hypothetical protein BRADI_4g31150v3

Cluster-12329.56429	-2.7	-2.2	-1.5	-2.6	hypothetical protein BRADI_5g03327v3
Cluster-12329.23994	2.2	2.1	2.8	4.0	hypothetical protein CUMW_252510, partial
Cluster-17139.0	-7.9	-7.8	-5.4	-5.4	hypothetical protein DD237_003955
Cluster-27360.1	-3.2	-6.5	-4.3	-5.2	hypothetical protein DYB32_000658
Cluster-12329.28366	-2.1	-1.2	-1.5	-2.5	hypothetical protein GQ55_2G049000
Cluster-12329.15541	2.5	2.8	1.9	3.0	hypothetical protein GQ55_5G192500
Cluster-12329.43996	3.6	4.5	4.0	6.2	hypothetical protein GQ55_8G157800
Cluster-12329.37837	4.4	5.3	5.5	7.5	hypothetical protein GQ55_9G600100
Cluster-12329.68880	2.1	2.7	2.8	4.1	hypothetical protein OsI_14820
Cluster-12329.57293	3.6	2.6	1.9	1.9	hypothetical protein OsJ_18467
Cluster-27955.0	-6.3	-7.2	-6.2	-6.2	hypothetical protein SELMODRAFT_419864
Cluster-12329.11108	4.1	4.5	2.8	4.1	hypothetical protein TRIUR3_02712
Cluster-12329.67466	2.9	3.1	2.5	3.9	hypothetical protein TRIUR3_04131
Cluster-12329.16582	6.1	5.9	3.3	5.4	hypothetical protein TRIUR3_14005
Cluster-12329.27476	-2.2	-1.6	-2.6	-3.5	hypothetical protein TRIUR3_18039
Cluster-12329.17145	6.8	6.4	4.9	7.3	hypothetical protein TRIUR3_24891
Cluster-12329.18011	2.5	2.3	3.1	3.7	jacalin-related lectin 19-like, partial
Cluster-12329.53964	2.8	4.0	4.4	6.7	late embryogenesis abundant protein 1
Cluster-12329.53965	3.4	4.3	4.5	6.8	late embryogenesis abundant protein, group 3-like
Cluster-12329.27453	4.4	5.1	3.1	5.6	late embryogenesis abundant protein, group 3-like
Cluster-12329.70282	5.6	5.8	5.5	7.9	late embryogenesis abundant protein, group 3-like
Cluster-12329.54680	3.2	3.0	2.8	3.7	linoleate 9S-lipoxygenase 2-like
Cluster-12329.30862	-1.5	-2.3	-2.8	-4.3	NAC domain-containing protein 21/22-like
Cluster-12329.59605	1.7	2.0	2.2	4.0	NADP-dependent malic enzyme
Cluster-12329.52489	-3.2	-2.8	-1.3	-2.0	nucleolar protein 58-like
Cluster-12329.73520	3.5	3.0	1.4	3.3	oleosin 1-like
Cluster-12329.59601	-3.2	-2.3	-3.3	-5.9	Peroxidase 15
Cluster-12329.33694	2.8	4.3	1.4	2.9	phytoene synthase 2, chloroplastic-like
Cluster-12329.15714	3.2	4.1	4.6	6.9	plasma membrane associated protein-1
Cluster-12329.73210	3.0	2.9	1.9	3.9	potassium channel KOR1-like isoform X1
Cluster-12329.82383	3.6	4.0	4.2	6.7	predicted protein
Cluster-30628.0	-6.6	-6.6	-3.5	-6.2	predicted protein
Cluster-12329.28775	3.8	3.2	2.0	3.5	predicted protein
Cluster-12329.12011	4.6	5.3	2.4	5.0	predicted protein
Cluster-12329.69415	5.2	3.5	1.8	2.5	predicted protein
Cluster-12329.38069	-1.3	-1.6	-1.3	-2.0	predicted protein
Cluster-12329.40903	-2.6	-2.2	-3.8	-4.6	predicted protein
Cluster-12329.5387	-2.5	-2.0	-2.9	-4.5	predicted protein
Cluster-12329.74926	4.9	4.5	2.9	3.9	predicted protein
Cluster-12329.69554	3.6	3.5	4.9	6.8	predicted protein
Cluster-12329.68697	5.9	6.1	5.1	7.8	predicted protein
Cluster-12329.15409	4.1	4.0	4.2	6.1	predicted protein
Cluster-12329.13801	4.3	6.5	4.7	8.1	predicted protein
Cluster-12329.60164	-2.5	-2.3	-1.5	-3.0	predicted protein
Cluster-12329.19357	2.4	2.8	1.8	2.8	predicted protein
Cluster-12329.77573	-8.1	-5.2	-3.2	-5.4	predicted protein
Cluster-12329.29960	2.1	2.0	1.4	2.4	predicted protein
Cluster-12329.19255	3.9	3.9	3.7	5.6	predicted protein

Cluster-12329.14664	3.4	5.0	2.1	4.8	predicted protein
Cluster-12329.31089	3.2	2.4	1.9	2.2	predicted protein, partial
Cluster-12329.59227	-1.4	-1.1	-2.6	-3.5	probable calcium-transporting ATPase 6, plasma membrane-type
Cluster-12329.12846	2.3	2.6	1.6	3.1	probable fucosyltransferase 8
Cluster-12329.39746	-2.1	-1.6	-1.9	-2.5	probable LRR receptor-like serine/threonine-protein kinase At1g56140
Cluster-12329.50890	-1.5	-1.2	-1.7	-2.5	probable LRR receptor-like serine/threonine-protein kinase At1g56140
Cluster-12329.72876	-1.6	-1.7	-2.3	-2.9	probable LRR receptor-like serine/threonine-protein kinase At3g47570 isoform X1
Cluster-12329.57952	2.8	3.5	1.4	2.8	probable protein phosphatase 2C 50
Cluster-12329.13918	2.5	3.0	2.5	4.1	probable protein phosphatase 2C 8
Cluster-12329.54528	1.9	2.3	1.9	2.2	probable sucrose-phosphate synthase 5
Cluster-12329.19483	4.7	5.5	4.5	7.0	Protein LE25
Cluster-12329.53849	2.0	1.3	2.2	3.0	protein RETICULATA-RELATED 5, chloroplastic-like
Cluster-12329.70665	3.5	4.8	1.9	3.9	putative clathrin assembly protein
Cluster-12329.53530	-1.2	-1.3	-1.3	-1.5	putative disease resistance protein RGA3
Cluster-12329.12147	4.1	4.5	4.0	4.6	Putative invertase inhibitor
Cluster-12329.26350	-2.1	-2.4	-1.8	-2.8	putative receptor-like protein kinase At4g00960 isoform X1
Cluster-12329.54104	-2.0	-2.1	-1.9	-3.8	PYL3
Cluster-12329.70108	2.5	2.8	2.3	3.3	pyruvate decarboxylase 1-like
Cluster-12329.19311	2.3	2.6	2.2	3.9	retrotransposon protein, putative, Ty3-gypsy subclass
Cluster-12329.19313	1.7	2.1	1.3	2.0	retrotransposon protein, putative, Ty3-gypsy subclass
Cluster-12329.39137	-1.6	-1.1	-1.2	-1.4	Rp1-like protein
Cluster-12329.25017	-2.3	-2.4	-2.5	-2.7	SnTox1 sensitivity protein
Cluster-12329.29018	3.1	3.7	3.1	4.7	sucrose synthase 4
Cluster-12329.29020	3.0	3.5	3.0	4.6	sucrose synthase 4
Cluster-12329.21992	2.8	3.1	1.3	2.4	TB2/DP1 protein
Cluster-12329.17299	5.1	6.0	4.1	7.2	translocator protein homolog
Cluster-12329.74266	4.1	6.7	2.9	5.8	uncharacterized protein LOC100828693
Cluster-12329.42771	2.4	2.8	1.4	3.0	uncharacterized protein LOC100837178
Cluster-12329.43214	-1.5	-2.1	-2.8	-3.3	uncharacterized protein LOC104584952
Cluster-12329.48150	3.8	3.1	4.4	5.6	uncharacterized protein LOC109716535 isoform X1
Cluster-12329.17539	4.7	5.8	3.3	6.2	uncharacterized protein LOC109732782
Cluster-12329.83151	3.5	4.5	3.4	6.2	uncharacterized protein LOC109745464
Cluster-12329.69103	5.3	6.5	3.3	5.6	uncharacterized protein LOC109752199
Cluster-12329.70769	3.7	3.5	1.7	3.2	uncharacterized protein LOC109753512
Cluster-12329.18408	3.0	3.1	2.7	3.9	uncharacterized protein LOC109753512
Cluster-12329.19760	5.2	5.5	3.2	6.0	uncharacterized protein LOC109754768
Cluster-12329.30953	3.7	3.4	4.1	6.1	uncharacterized protein LOC109762444
Cluster-12329.14413	3.1	4.0	1.5	3.5	uncharacterized protein LOC109767342
Cluster-12329.61249	1.9	1.9	1.3	2.0	uncharacterized protein LOC109772703
Cluster-12329.19134	4.5	5.3	3.2	5.5	uncharacterized protein LOC109773736
Cluster-12329.70583	6.1	6.6	3.8	6.3	uncharacterized protein LOC4331521
Cluster-19103.0	-6.2	-7.1	-5.7	-6.6	unknown

Cluster-12329.64076	-4.4	-6.8	-4.1	-5.1	unknown
Cluster-12329.27244	2.4	2.6	2.1	3.3	unnamed protein product
Cluster-12329.40591	3.1	2.3	2.1	2.9	unnamed protein product
Cluster-12329.65650	2.4	2.7	1.6	3.1	unnamed protein product
Cluster-12329.19319	4.3	4.2	3.6	5.7	unnamed protein product
Cluster-12329.20262	3.5	4.0	1.5	3.1	unnamed protein product
Cluster-12329.15018	-2.5	-2.1	-2.9	-5.6	unnamed protein product
Cluster-12329.69497	4.9	4.0	4.5	5.7	Vicilin-like antimicrobial peptides 2-2
Cluster-12329.10917	3.2	5.0	3.4	5.9	V-type proton ATPase subunit D-like
Cluster-12329.70389	2.4	3.2	2.6	2.5	wheatwin-2

Note: The screening criteria for specifically expressed genes are |log2FC|>1, p<0.05.

Table S2. The specifically expresses of JIA2 differential genes under drought stress.

Gene id	JIA2-1 fpkm	JIA2-2 fpkm	JIA2-2 log2FC	JIA2-3 fpkm	JIA2-3 log2FC	Description
Cluster-12329.52838	9.9	649.1	5.9	549.6	5.7	--
Cluster-12329.76640	40.6	2.3	-4.2	1.8	-4.7	--
Cluster-12329.46046	3.2	154.3	5.5	395.2	6.8	--
Cluster-12329.52286	50.2	6.5	-3.1	6.3	-3.1	--
Cluster-12329.44964	250.5	33.8	-3.0	22.5	-3.6	1-aminocyclopropane-1-carboxylate oxidase-like
Cluster-12329.48023	176.2	23.4	-3.0	14.5	-3.7	1-aminocyclopropane-1-carboxylate oxidase-like
Cluster-12329.49732	392.4	48.5	-3.1	28.5	-3.9	ACC oxidase
Cluster-12329.49731	284.3	32.9	-3.2	19.2	-4.0	ACC oxidase
Cluster-12329.27503	30.4	2.6	-3.7	2.7	-3.6	aspartyl protease family protein At5g10770-like
Cluster-12329.55589	42.7	3.0	-4.0	2.6	-4.2	cysteine-rich receptor-like protein kinase 10
Cluster-12329.55590	31.3	3.7	-3.2	2.0	-4.1	cysteine-rich receptor-like protein kinase 10
Cluster-12329.22590	118.5	12.4	-3.4	4.7	-4.8	dirigent protein 5-like
Cluster-12329.40430	61.3	5.2	-3.7	2.2	-4.9	ethylene response factor
Cluster-12329.33234	60.6	7.4	-3.2	4.7	-3.8	glucan endo-1,3-beta-glucosidase 3-like isoform X1
Cluster-12329.33233	40.2	5.2	-3.1	3.7	-3.6	glucan endo-1,3-beta-glucosidase 3-like isoform X1
Cluster-12329.25273	64.7	7.9	-3.2	2.5	-4.8	heme-binding-like protein At3g10130, chloroplastic
Cluster-12329.8036	126.0	12.5	-3.4	2.6	-5.7	hypothetical protein BRADI_2g47510v3
Cluster-12329.56709	39.0	4.8	-3.1	3.4	-3.6	hypothetical protein BRADI_4g31430v3
Cluster-12329.74492	173.8	11.7	-4.0	7.0	-4.8	hypothetical protein OsI_20854
Cluster-12329.54760	104.1	11.9	-3.2	7.6	-3.9	hypothetical protein TRIUR3_01630
Cluster-12329.38808	32.3	4.1	-3.1	2.0	-4.2	hypothetical protein TRIUR3_31101
Cluster-12329.26155	151.9	17.8	-3.2	6.3	-4.7	IQ domain-containing protein IQM1
Cluster-12329.28038	40.9	461.8	3.4	683.5	3.9	metallothionein-like protein type 2
Cluster-12329.18219	150.5	16.1	-3.3	7.9	-4.4	mitogen-activated protein kinase kinase kinase 3-like
Cluster-12329.51406	152.6	19.1	-3.1	9.4	-4.2	phenylalanine ammonia-lyase 1
Cluster-12329.51405	134.3	15.4	-3.2	8.5	-4.1	phenylalanine ammonia-lyase 2
Cluster-12329.33610	60.7	7.3	-3.2	2.3	-4.9	predicted protein

Cluster-12329.77790	20.5	1.9	-3.5	2.2	-3.3	predicted protein
Cluster-12329.10176	49.8	6.3	-3.1	4.3	-3.7	predicted protein
Cluster-12329.32862	167.9	20.2	-3.2	10.1	-4.2	predicted protein
Cluster-12329.40903	26.8	2.0	-3.8	1.2	-4.6	predicted protein
Cluster-12329.55591	23.7	2.4	-3.4	1.3	-4.3	predicted protein
Cluster-12329.62594	4.4	90.1	4.2	192.6	5.3	predicted protein
Cluster-12329.51932	162.6	13.2	-3.8	18.7	-3.3	predicted protein
Cluster-12329.68011	79.4	4.2	-4.3	2.8	-5.0	predicted protein, partial
Cluster-12329.24188	217.1	27.7	-3.1	2.8	-6.4	predicted protein, partial
Cluster-12329.46439	196.6	26.2	-3.0	18.9	-3.5	PREDICTED: cationic peroxidase SPC4-like
Cluster-12329.65038	54.8	7.2	-3.1	4.4	-3.8	probable calcium-binding protein CML10
Cluster-12329.54015	84.5	6.6	-3.8	5.5	-4.1	probable carboxylesterase 15
Cluster-12329.54017	70.2	6.8	-3.5	4.6	-4.1	probable carboxylesterase 15
Cluster-12329.53604	131.3	11.6	-3.6	5.4	-4.7	probable WRKY transcription factor 70
Cluster-12329.66018	396.5	40.6	-3.4	19.2	-4.5	protein TIFY 11e-like
Cluster-12329.66019	182.6	19.7	-3.3	8.5	-4.6	protein TIFY 11e-like
Cluster-12329.53548	57.4	7.4	-3.1	6.9	-3.2	putative acyl transferase 6
Cluster-12329.55264	69.5	9.3	-3.0	2.0	-5.2	Putative disease resistance RPP13-like protein 1
Cluster-12329.24192	88.8	11.9	-3.0	2.1	-5.6	putative WRKY transcription factor 46
Cluster-12329.44119	93.5	12.3	-3.0	12.6	-3.0	Q-type C2H2 zinc finger protein
Cluster-12329.23063	81.4	8.8	-3.3	3.1	-4.9	RING-H2 finger protein ATL3-like
Cluster-12329.23854	45.4	5.1	-3.3	3.1	-4.0	serine/threonine-protein kinase RIPK
Cluster-12329.30337	31.3	4.1	-3.0	1.8	-4.2	U-box domain-containing protein 27
Cluster-12329.10245	35.3	1.7	-4.5	1.7	-4.5	uncharacterized protein LOC109741409
Cluster-12329.7920	65.7	7.1	-3.3	5.6	-3.7	uncharacterized protein LOC109765335
Cluster-12329.7921	37.1	4.2	-3.3	1.5	-4.8	uncharacterized protein LOC109765335
Cluster-12329.23823	28.4	350.5	3.5	519.5	4.0	uncharacterized protein LOC109783551
Cluster-12329.40403	6.0	57.5	3.1	55.5	3.1	uncharacterized protein LOC109783551
Cluster-12329.9976	79.3	8.2	-3.4	2.3	-5.3	uncharacterized protein LOC109784088
Cluster-12329.32861	113.7	12.1	-3.3	4.6	-4.8	unnamed protein product
Cluster-12329.61828	18.7	2.1	-3.3	2.2	-3.2	unnamed protein product
Cluster-12329.39006	21.0	2.8	-3.0	1.7	-3.8	wall-associated receptor kinase 2-like

Note: The screening criteria for specifically expressed genes(up-regulated and down-regulated) are $|log2FC|>3$, $p<0.05$.

Table S3. The co-express differential proteins of BA9 and JIA2 under drought stress.

Gene id	Description	BA9-2/9-1		BA9-3/9-1		JIA2-2/2-1		JIA2-3/2-1	
		FC	p-value	FC	p-value	FC	p-value	FC	p-value
Cluster-12329.42730	12-oxo-phytodienoic acid reductase 2	0.457	0.002	0.564	0.004	0.568	0.001	0.530	0.000
Cluster-12329.40415	26 kDa endochitinase 1-like	3.224	0.000	2.921	0.001	2.143	0.042	2.809	0.009
Cluster-12329.39510	ABC transporter G family member 48-like	2.081	0.002	1.828	0.020	0.985	0.973	1.140	0.781

Cluster-12329.30175	Adipocyte plasma membrane-associated protein	3.083	0.000	1.724	0.001	0.682	0.027	0.603	0.013
Cluster-12329.40387	alpha-galactosidase-like	0.656	0.017	1.556	0.024	1.160	0.742	1.468	0.305
Cluster-12329.42859	ammonium transporter AMT2.1	0.378	0.004	0.456	0.011	0.120	0.108	0.134	0.110
Cluster-12329.58512	anthranilate synthase alpha 2 subunit	0.348	0.004	0.521	0.003	0.590	0.005	0.421	0.002
Cluster-12329.42873	ATP-citrate synthase alpha chain protein 2	3.520	0.002	2.604	0.011	1.428	0.448	1.520	0.449
Cluster-12329.25236	berberine bridge enzyme-like 27	0.356	0.000	0.555	0.007	0.310	0.001	0.282	0.001
Cluster-12329.46928	beta-glucosidase 4	1.761	0.011	2.979	0.035	1.433	0.303	1.829	0.238
Cluster-12329.48569	cinnamyl alcohol dehydrogenase	0.369	0.041	0.542	0.031	0.821	0.352	0.733	0.229
Cluster-12329.46789	cytochrome P450	0.548	0.047	0.674	0.024	0.566	0.051	0.612	0.075
Cluster-12329.48221	cytochrome P450	1.656	0.012	1.428	0.036	0.304	0.033	0.294	0.032
Cluster-12329.18884	cytochrome P450 CYP99A1-like	2.200	0.011	2.632	0.018	2.218	0.003	1.555	0.286
Cluster-12329.53808	cytosolic Cu/Zn superoxide dismutase	0.770	0.044	1.424	0.025	1.341	0.002	1.381	0.006
Cluster-12329.22699	dicarboxylate transporter 2.1, chloroplastic	0.611	0.038	0.627	0.028	1.170	0.544	1.766	0.220
Cluster-12329.47448	E3 ubiquitin-protein ligase KEG	2.610	0.001	3.691	0.048	1.191	0.158	0.971	0.872
Cluster-12329.38222	electron transfer flavoprotein-ubiquinone oxidoreductase, mitochondrial isoform X3	0.459	0.048	0.478	0.038	2.809	0.002	2.571	0.001
Cluster-12329.47209	elongation factor-like GTPase 1	1.791	0.040	1.483	0.040	1.080	0.569	1.416	0.119
Cluster-12329.43587	fructan exohydrolase	0.664	0.026	0.503	0.010	0.284	0.000	0.234	0.000
Cluster-12329.44239	hydroxyanthranilate hydroxycinnamoyltr ansferase 3	0.768	0.031	0.555	0.001	0.544	0.050	1.012	0.982
Cluster-12329.29762	hypothetical protein BRADI_1g07910v3	1.580	0.009	1.414	0.028	1.515	0.008	1.340	0.086
Cluster-12329.45583	hypothetical protein BRADI_2g08250v3	0.816	0.040	0.727	0.046	0.560	0.001	0.487	0.000
Cluster-12329.46014	hypothetical protein BRADI_2g42380v3	1.449	0.012	1.738	0.035	1.145	0.729	1.515	0.376
Cluster-12329.54345	hypothetical protein BRADI_2g44856v3	1.685	0.002	1.802	0.002	0.879	0.498	0.909	0.598

Cluster-12329.76277	hypothetical protein BRADI_2g52317v3	1.974	0.024	1.786	0.048	1.259	0.505	1.396	0.511
Cluster-12329.49150	hypothetical protein BRADI_4g08097v3	0.647	0.004	0.601	0.004	0.672	0.065	0.588	0.018
Cluster-12329.70711	hypothetical protein C2845_PM01G12550	2.075	0.001	1.812	0.012	0.469	0.242	0.856	0.731
Cluster-12329.57642	hypothetical protein OsI_09291	1.758	0.002	1.378	0.029	1.135	0.353	1.321	0.074
Cluster-12329.47167	hypothetical protein OsI_16658	0.473	0.015	0.523	0.023	0.435	0.018	0.566	0.095
Cluster-12329.46264	NADH--cytochrome b5 reductase 1	0.772	0.006	0.752	0.006	0.663	0.026	0.561	0.008
Cluster-12329.40903	papain-like cysteine proteinase	0.083	0.014	0.106	0.003	0.336	0.068	0.308	0.060
Cluster-12329.42451	phospho-2-dehydro-3-deoxyheptonate aldolase 1, chloroplastic	0.390	0.025	0.466	0.007	0.808	0.402	0.745	0.300
Cluster-12329.45920	phospholipid-transporting ATPase 3 isoform X1	0.315	0.001	0.293	0.000	0.913	0.563	1.120	0.253
Cluster-12329.53668	plastid glutamine synthetase isoform GS2b	2.244	0.001	1.760	0.017	0.686	0.006	0.762	0.038
Cluster-12329.47790	polygalacturonase inhibitor	1.994	0.019	1.450	0.050	1.967	0.001	1.828	0.151
Cluster-12329.47853	polyol transporter 5-like	2.947	0.030	2.418	0.035	1.021	0.886	1.154	0.316
Cluster-12329.43728	predicted protein	1.400	0.019	1.475	0.035	0.821	0.253	0.750	0.107
Cluster-12329.45053	predicted protein	1.322	0.020	1.722	0.005	0.676	0.072	0.491	0.022
Cluster-12329.64971	predicted protein	0.353	0.007	0.584	0.022	0.464	0.091	0.577	0.287
Cluster-12329.45487	predicted protein	0.264	0.011	0.574	0.012	0.452	0.005	0.577	0.024
Cluster-12329.58094	predicted protein	0.390	0.049	0.528	0.046	0.668	0.175	0.886	0.763
Cluster-12329.50128	predicted protein	0.607	0.003	0.622	0.048	0.999	0.996	0.635	0.050
Cluster-12329.29002	predicted protein	2.032	0.018	1.662	0.048	1.854	0.017	1.761	0.033
Cluster-12329.19503	predicted protein, partial	1.585	0.007	1.277	0.020	0.899	0.739	0.601	0.215
Cluster-12329.50406	succinyl-CoA ligase subunit beta, mitochondrial	1.159	0.012	1.235	0.035	0.810	0.067	0.773	0.047
Cluster-12329.44784	PREDICTED: sulfite oxidase	0.448	0.002	0.561	0.006	0.960	0.805	0.635	0.127
Cluster-12329.44784	PREDICTED: sulfite oxidase	0.754	0.037	0.698	0.026	0.909	0.066	0.803	0.038
Cluster-12329.27553	putativeUDP-rhamnose:rhamnosyl transferase 1	0.624	0.042	0.369	0.002	0.997	0.990	1.031	0.910
Cluster-12329.21291	pyridoxine/pyridoxa mine 5-phosphate	1.300	0.037	0.333	0.001	1.011	0.689	1.060	0.359

	oxidase 1, chloroplastic								
Cluster-12329.45221	S-adenosyl-L-homocysteine hydrolase	0.596	0.016	0.670	0.006	0.989	0.920	1.004	0.963
Cluster-12329.47122	subtilisin-like protease SBT1.7	0.447	0.006	0.679	0.023	0.620	0.176	0.394	0.083
Cluster-12329.33253	Threonine synthase 1, chloroplastic	1.474	0.001	1.264	0.031	1.787	0.000	1.557	0.099
Cluster-12329.58040	UDP-glycosyltransferase 72B1-like	0.335	0.014	0.471	0.007	0.279	0.002	0.200	0.015
Cluster-12329.39344	UGT80A24	2.163	0.001	1.986	0.001	0.790	0.058	0.980	0.909
Cluster-12329.23406	uncharacterized protein LOC100841867	0.320	0.042	0.398	0.026	0.339	0.255	1.033	0.957
Cluster-12329.37542	uncharacterized protein LOC109769744	1.988	0.004	1.349	0.033	1.295	0.207	2.294	0.077
Cluster-12329.45937	uncharacterized protein LOC109775963	1.316	0.003	1.287	0.005	1.049	0.399	1.168	0.015
Cluster-12329.46196	uncharacterized protein LOC541714 isoform X2	0.427	0.028	0.504	0.008	0.809	0.273	0.878	0.493
Cluster-12329.17719	universal stress protein PHOS32-like	0.529	0.005	0.543	0.010	0.543	0.007	0.411	0.003
Cluster-12329.41351	unnamed protein product	0.415	0.002	0.515	0.033	0.529	0.058	0.533	0.062
Cluster-12329.71832	unnamed protein product	3.554	0.001	4.595	0.003	2.040	0.172	3.491	0.001
Cluster-12329.46829	V-type proton ATPase catalytic subunit A	1.952	0.001	1.462	0.043	0.964	0.725	0.805	0.005

Table S4. The specifically expresses differential proteins of JIA2 under drought stress.

Protein	JIA2-2/2-1 FC	p-value	JIA2-2/2-1 FC	p-value	GO/KEGG Description
Cluster-12329.48386	0.41	0.0128	0.37	0.0071	xyloglucan galactosyltransferase MUR3
Cluster-12329.14848	0.45	0.0225	0.47	0.0253	xylan biosynthetic process
Cluster-12329.36881	2.12	0.0163	2.41	0.0127	vacuolar protein sorting-associated protein 54
Cluster-12329.43629	0.40	0.0257	0.44	0.0279	urease accessory protein
Cluster-12329.46978	2.87	0.0091	3.27	0.0029	tRNA guanosine-2'-O-methyltransferase
Cluster-12329.39756	0.48	0.0049	0.47	0.0289	transporter activity
Cluster-12329.42106	0.44	0.0054	0.38	0.0017	transporter activity
Cluster-12329.45904	0.40	0.0262	0.33	0.0341	transporter activity
Cluster-12329.46297	0.40	0.0311	0.42	0.0077	transporter activity
Cluster-12329.46729	0.28	0.0025	0.24	0.0022	transporter activity
Cluster-12329.39937	0.44	0.0448	0.45	0.0448	transport
Cluster-12329.44647	0.36	0.0029	0.30	0.0007	transport

Cluster-12329.52083	0.50	0.0191	0.36	0.0094	transport
Cluster-12329.57154	2.49	0.0254	2.52	0.0062	transmembrane transport
Cluster-12329.39132	2.82	0.0022	2.76	0.0001	transketolase
Cluster-12329.33100	3.04	0.0000	2.49	0.0022	transaminase activity
Cluster-12329.36721	3.16	0.0489	4.92	0.0004	trafficking protein particle complex subunit 9
Cluster-12329.50073	2.32	0.0116	2.35	0.0058	threonine-type endopeptidase activity
Cluster-12329.56189	2.54	0.0360	2.83	0.0017	terpenoid biosynthetic process
Cluster-12329.59076	7.07	0.0000	11.86	0.0341	telomere maintenance
Cluster-12329.41271	0.32	0.0002	0.50	0.0021	sulfotransferase activity
Cluster-12329.38036	3.37	0.0376	3.03	0.0293	structural constituent of ribosome
Cluster-11888.0	2.32	0.0105	2.92	0.0013	structural constituent of ribosome
Cluster-12329.19614	2.05	0.0056	2.45	0.0030	structural constituent of ribosome
Cluster-12329.25578	2.51	0.0178	2.98	0.0225	structural constituent of ribosome
Cluster-12329.45480	2.97	0.0053	3.28	0.0298	structural constituent of ribosome
Cluster-12329.47059	2.18	0.0030	2.05	0.0220	structural constituent of ribosome
Cluster-12329.47776	2.20	0.0031	2.10	0.0135	structural constituent of ribosome
Cluster-12329.47836	2.64	0.0033	2.85	0.0408	structural constituent of ribosome
Cluster-12329.48202	3.17	0.0002	3.31	0.0082	structural constituent of ribosome
Cluster-12329.50106	3.38	0.0044	3.55	0.0090	structural constituent of ribosome
Cluster-12329.53055	2.33	0.0010	2.53	0.0029	structural constituent of ribosome
Cluster-12329.59685	4.38	0.0001	5.86	0.0005	structural constituent of ribosome
Cluster-12329.69623	5.79	0.0079	5.70	0.0031	structural constituent of ribosome
Cluster-16534.0	5.81	0.0001	7.93	0.0001	structural constituent of ribosome
Cluster-27122.0	2.08	0.0036	2.36	0.0131	structural constituent of ribosome
Cluster-12329.47699	0.34	0.0025	0.31	0.0025	structural constituent of cytoskeleton
Cluster-12329.60172	3.50	0.0099	3.37	0.0047	splicing factor, arginine/serine-rich 7
Cluster-12329.26481	2.28	0.0025	2.05	0.0057	spartin
Cluster-12329.35552	2.45	0.0146	2.30	0.0196	seryl-tRNA synthetase
Cluster-12329.35428	3.84	0.0005	6.42	0.0007	serine-type endopeptidase inhibitor activity
Cluster-12329.60990	3.15	0.0265	2.65	0.0318	serine-type endopeptidase inhibitor activity
Cluster-12329.48183	2.82	0.0136	2.95	0.0115	serine-type endopeptidase activity
Cluster-12329.49419	0.17	0.0002	0.29	0.0172	serine-type endopeptidase activity
Cluster-12329.38038	4.41	0.0236	4.96	0.0030	serine-type carboxypeptidase activity
Cluster-12329.53402	0.32	0.0205	0.27	0.0030	serine-type carboxypeptidase activity
Cluster-12329.43182	3.14	0.0463	3.70	0.0111	sequence-specific DNA binding
Cluster-12329.27710	2.56	0.0487	2.82	0.0174	SAP domain-containing ribonucleoprotein
Cluster-12329.52048	3.44	0.0002	3.86	0.0007	RNA processing
Cluster-12329.50126	4.30	0.0192	4.98	0.0090	RNA methylation
Cluster-12329.42822	2.09	0.0078	2.59	0.0029	RNA binding
Cluster-12329.21735	2.42	0.0011	2.64	0.0110	ribosome biogenesis protein BRX1
Cluster-12329.46311	3.19	0.0024	2.86	0.0054	ribosomal RNA-processing protein 12
Cluster-12329.51283	2.53	0.0011	2.67	0.0031	regulation of translation
Cluster-2168.0	0.21	0.0003	0.17	0.0021	pyroglutamyl-peptidase
Cluster-12329.62295	3.15	0.0046	3.02	0.0250	pseudouridine synthesis
Cluster-12329.58022	2.34	0.0055	2.98	0.0139	proton-transporting ATP synthase complex assembly
Cluster-12329.43644	0.28	0.0155	0.11	0.0010	proteolysis
Cluster-12329.35349	3.24	0.0096	3.24	0.0003	proteolysis

Cluster-12329.43809	0.44	0.0053	0.38	0.0119	proteolysis
Cluster-12329.45109	0.34	0.0022	0.32	0.0026	proteolysis
Cluster-22035.1	0.34	0.0063	0.25	0.0182	proteolysis
Cluster-12329.31876	0.36	0.0093	0.47	0.0203	protein kinase activity
Cluster-12329.46753	0.45	0.0032	0.37	0.0049	protein kinase activity
Cluster-12329.50363	0.50	0.0006	0.48	0.0014	protein kinase activity
Cluster-12329.58642	0.18	0.0202	0.28	0.0347	protein kinase activity
Cluster-12329.45749	0.36	0.0000	0.39	0.0000	protein domain specific binding
Cluster-21280.0	4.95	0.0003	4.78	0.0000	protein dimerization activity
Cluster-12329.41964	0.42	0.0139	0.38	0.0064	protein binding
Cluster-12329.50319	0.38	0.0049	0.24	0.0020	protein binding
Cluster-12329.15022	3.21	0.0135	3.11	0.0323	protein binding
Cluster-12329.16346	2.42	0.0066	3.31	0.0017	protein binding
Cluster-12329.22341	3.19	0.0323	4.47	0.0000	protein binding
Cluster-12329.34668	3.41	0.0022	5.42	0.0006	protein binding
Cluster-12329.39776	3.32	0.0024	2.71	0.0066	protein binding
Cluster-12329.42509	0.37	0.0014	0.37	0.0404	protein binding
Cluster-12329.46138	0.42	0.0344	0.32	0.0298	protein binding
Cluster-12329.46986	4.39	0.0244	3.75	0.0472	protein binding
Cluster-12329.47845	0.29	0.0019	0.29	0.0046	protein binding
Cluster-12329.51162	2.29	0.0051	2.11	0.0135	protein binding
Cluster-12329.54023	2.00	0.0265	2.35	0.0458	protein binding
Cluster-12329.55764	3.46	0.0155	5.60	0.0231	protein binding
Cluster-12329.61573	0.19	0.0001	0.17	0.0001	protein binding
Cluster-12329.40181	0.29	0.0394	0.27	0.0377	prenylcysteine oxidase activity
Cluster-30010.0	4.93	0.0005	3.71	0.0145	phosphoglycerate mutase activity
Cluster-12329.33490	2.19	0.0038	2.63	0.0080	peroxin-5
Cluster-12329.14980	0.47	0.0025	0.30	0.0066	peroxidase activity
Cluster-12329.33750	0.49	0.0092	0.32	0.0065	peroxidase activity
Cluster-12329.36977	0.39	0.0007	0.30	0.0004	peroxidase activity
Cluster-12329.40101	0.12	0.0205	0.11	0.0032	peroxidase activity
Cluster-12329.42981	0.35	0.0040	0.28	0.0019	peroxidase activity
Cluster-12329.43312	0.40	0.0020	0.42	0.0111	peroxidase activity
Cluster-12329.43857	0.35	0.0195	0.30	0.0147	peroxidase activity
Cluster-12329.52051	0.28	0.0004	0.26	0.0004	peroxidase activity
Cluster-12329.52052	0.23	0.0064	0.23	0.0023	peroxidase activity
Cluster-12329.59924	0.44	0.0005	0.36	0.0026	peroxidase activity
Cluster-27561.0	0.34	0.0071	0.24	0.0039	peroxidase activity
Cluster-12329.73097	2.53	0.0027	2.16	0.0023	oxygen binding
Cluster-12329.45913	0.31	0.0057	0.26	0.0119	oxidoreductase activity
Cluster-12329.46857	0.49	0.0348	0.48	0.0075	oxidoreductase activity
Cluster-12329.49670	0.41	0.0042	0.36	0.0040	oxidoreductase activity
Cluster-12329.61877	0.48	0.0024	0.25	0.0003	oxidoreductase activity
Cluster-12329.60712	2.80	0.0073	2.50	0.0206	oxidation-reduction process
Cluster-12329.61816	0.36	0.0468	0.12	0.0183	omega-hydroxypalmitate O-feruloyl transferase
Cluster-12329.69497	6.09	0.0004	6.37	0.0302	nutrient reservoir activity
Cluster-12329.58725	0.44	0.0020	0.34	0.0091	nucleotide binding
Cluster-12329.45303	2.94	0.0025	2.37	0.0370	nucleosome
Cluster-22588.0	2.80	0.0306	3.57	0.0002	nucleosome
Cluster-12329.48442	2.19	0.0020	2.23	0.0063	nucleolar protein 56

Cluster-3608.0	9.51	0.0011	8.52	0.0244	nucleic acid binding
Cluster-12329.17354	2.50	0.0384	2.66	0.0020	nucleic acid binding
Cluster-12329.33078	2.48	0.0066	2.59	0.0049	nucleic acid binding
Cluster-12329.42921	2.03	0.0201	2.68	0.0052	nucleic acid binding
Cluster-12329.56144	2.22	0.0298	2.19	0.0301	nucleic acid binding
Cluster-12329.30577	2.29	0.0270	2.41	0.0255	nuclear pore complex protein Nup85
Cluster-12329.45111	2.90	0.0094	2.56	0.0402	nuclear GTP-binding protein
Cluster-12329.42303	0.27	0.0005	0.41	0.0415	nicotinate phosphoribosyltransferase
Cluster-12329.42372	0.18	0.0008	0.20	0.0007	negative regulation of translation
Cluster-25204.0	2.13	0.0204	2.12	0.0172	NADH-ubiquinone oxidoreductase chain 6
Cluster-12329.47768	0.46	0.0241	0.32	0.0112	NADH dehydrogenase (ubiquinone) 1 beta subcomplex subunit 9
Cluster-12329.46665	0.30	0.0005	0.33	0.0052	NAD(P)H dehydrogenase (quinone)
Cluster-12329.46242	0.25	0.0114	0.29	0.0150	N-acetylneuraminate 9-O-acetyltransferase
Cluster-12329.51060	3.29	0.0006	2.19	0.0064	motor activity
Cluster-12329.23694	2.34	0.0108	2.86	0.0202	Molecular Function:protein binding
Cluster-12329.24463	0.43	0.0018	0.21	0.0001	Molecular Function:protein binding
Cluster-12329.41740	2.37	0.0148	2.66	0.0203	Molecular Function:catalytic activity
Cluster-12329.11672	2.09	0.0137	2.97	0.0005	mitochondrial phosphate transporter
Cluster-12329.17513	2.48	0.0084	2.45	0.0335	mitochondrial outer membrane translocase complex
Cluster-12329.45277	0.15	0.0007	0.21	0.0042	methyltransferase activity
Cluster-12329.45724	0.28	0.0205	0.37	0.0333	methionine adenosyltransferase activity
Cluster-12329.43185	0.47	0.0006	0.45	0.0066	metal ion transport
Cluster-12329.58884	2.12	0.0401	2.23	0.0164	metal ion binding
Cluster-12329.61926	0.49	0.0486	0.44	0.0408	metal ion binding
Cluster-12329.51889	2.66	0.0318	3.77	0.0013	metabolic process
Cluster-12329.57912	2.71	0.0028	3.09	0.0000	metabolic process
Cluster-12329.57969	0.35	0.0057	0.39	0.0087	metabolic process
Cluster-12329.63524	3.83	0.0002	3.66	0.0002	metabolic process
Cluster-12329.63525	5.34	0.0006	3.92	0.0001	metabolic process
Cluster-12329.63525	4.70	0.0004	4.28	0.0008	metabolic process
Cluster-30262.1	0.35	0.0006	0.26	0.0005	metabolic process
Cluster-12329.19357	5.21	0.0195	3.97	0.0005	membrane
Cluster-27225.0	3.33	0.0329	2.16	0.0201	membrane
Cluster-12329.40661	0.20	0.0335	0.32	0.0265	manganese ion binding
Cluster-12329.48850	0.40	0.0213	0.28	0.0068	malate metabolic process
Cluster-12329.55547	0.43	0.0035	0.25	0.0026	malate dehydrogenase
Cluster-12329.59603	7.03	0.0008	5.81	0.0023	malate dehydrogenase
Cluster-12329.23725	2.08	0.0393	3.50	0.0012	lipid transport
Cluster-12329.39105	0.35	0.0043	0.17	0.0023	lipid metabolic process
Cluster-12329.42824	0.30	0.0000	0.23	0.0001	lipid metabolic process
Cluster-12329.35244	4.75	0.0329	7.16	0.0043	large subunit ribosomal protein L7e
Cluster-12329.41754	2.18	0.0023	2.62	0.0002	large subunit ribosomal protein L7Ae
Cluster-12329.41497	2.51	0.0092	3.07	0.0034	large subunit ribosomal protein L27Ae
Cluster-12329.39606	6.27	0.0198	5.48	0.0012	large subunit ribosomal protein L24e
Cluster-12329.59427	3.41	0.0007	3.00	0.0174	lactoylglutathione lyase
Cluster-12329.48942	0.41	0.0094	0.24	0.0051	iron ion binding
Cluster-12329.48221	0.30	0.0334	0.29	0.0319	iron ion binding

Cluster-12329.51058	2.38	0.0021	2.20	0.0075	iron ion binding
Cluster-12329.17299	5.56	0.0011	4.36	0.0059	integral component of membrane
Cluster-12329.23588	3.13	0.0362	3.72	0.0099	integral component of membrane
Cluster-12329.26605	2.22	0.0038	3.14	0.0041	integral component of membrane
Cluster-12329.39422	0.32	0.0016	0.29	0.0013	integral component of membrane
Cluster-12329.44929	0.42	0.0010	0.28	0.0011	integral component of membrane
Cluster-12329.46026	2.32	0.0151	2.77	0.0063	inorganic diphosphatase activity
Cluster-12329.50264	0.40	0.0351	0.40	0.0409	hydroxymethylglutaryl-CoA synthase
Cluster-12329.53567	2.02	0.0092	2.34	0.0057	hydrolase activity
Cluster-12329.43240	7.43	0.0074	4.12	0.0137	heat shock 70kDa protein
Cluster-12329.29526	2.61	0.0012	3.69	0.0029	H/ACA ribonucleoprotein complex subunit 2
Cluster-12329.45696	0.25	0.0307	0.23	0.0144	GTPase activity
Cluster-12329.45717	0.43	0.0443	0.31	0.0270	GTPase activity
Cluster-20139.0	0.50	0.0036	0.39	0.0089	GTPase activity
Cluster-12329.45312	0.39	0.0052	0.38	0.0044	GTP binding
Cluster-12329.47784	2.76	0.0087	3.12	0.0005	GTP binding
Cluster-12329.49630	0.41	0.0385	0.28	0.0173	GTP binding
Cluster-12329.15446	0.46	0.0411	0.18	0.0328	glutathione S-transferase
Cluster-12329.29554	0.46	0.0338	0.24	0.0198	FMN binding
Cluster-24056.0	0.24	0.0372	0.34	0.0136	FMN binding
Cluster-12329.36679	2.03	0.0084	2.13	0.0284	fatty acid metabolic process
Cluster-12329.68708	8.79	0.0003	6.95	0.0089	extracellular space
Cluster-12329.55067	0.12	0.0293	0.11	0.0281	exocyst
Cluster-12329.40027	4.64	0.0051	6.31	0.0056	essential nuclear protein 1
Cluster-12329.30167	0.40	0.0167	0.44	0.0110	endoplasmic reticulum
Cluster-12329.42162	0.47	0.0017	0.36	0.0100	endo-1,3(4)-beta-glucanase
Cluster-12329.32714	0.30	0.0068	0.11	0.0009	electron carrier activity
Cluster-12329.43095	0.37	0.0089	0.40	0.0095	electron carrier activity
Cluster-12329.45383	0.39	0.0020	0.22	0.0099	electron carrier activity
Cluster-12329.50014	0.34	0.0241	0.26	0.0176	electron carrier activity
Cluster-12329.51396	0.49	0.0416	0.33	0.0334	electron carrier activity
Cluster-12329.53251	0.40	0.0003	0.45	0.0005	electron carrier activity
Cluster-12329.63759	0.32	0.0305	0.18	0.0157	electron carrier activity
Cluster-12329.58928	2.58	0.0045	3.41	0.0285	DnaJ homolog subfamily C member 2
Cluster-12329.47118	2.34	0.0301	3.00	0.0497	DNA binding
Cluster-12329.50646	2.32	0.0000	2.08	0.0023	DNA binding
Cluster-12329.10108	6.05	0.0108	6.18	0.0076	defense response
Cluster-12329.32721	0.32	0.0024	0.14	0.0016	cytochrome-c oxidase activity
Cluster-12329.46264	0.44	0.0003	0.40	0.0014	cytochrome-b5 reductase
Cluster-12329.49622	0.46	0.0150	0.36	0.0035	cysteine synthase A
Cluster-12329.47550	2.02	0.0096	2.86	0.0032	cullin-associated NEDD8-dissociated protein
Cluster-12329.47555	7.02	0.0220	9.93	0.0054	chitinase activity
Cluster-12329.39096	2.81	0.0085	2.76	0.0042	chitinase
Cluster-12329.36403	0.42	0.0037	0.46	0.0425	cellular amino acid metabolic process
Cluster-12329.36404	0.27	0.0039	0.19	0.0046	cellular amino acid metabolic process
Cluster-12329.53200	0.32	0.0463	0.35	0.0467	catalytic activity
Cluster-12329.42675	0.50	0.0251	0.30	0.0185	catalytic activity
Cluster-12329.35194	0.16	0.0011	0.13	0.0014	catalytic activity
Cluster-12329.41051	0.39	0.0013	0.19	0.0001	catalytic activity

Cluster-12329.41231	0.47	0.0135	0.38	0.0334	catalytic activity
Cluster-12329.58241	2.97	0.0061	3.05	0.0088	catalytic activity
Cluster-12329.60324	12.28	0.0001	10.59	0.0001	catalytic activity
Cluster-12329.60324	6.31	0.0005	4.79	0.0030	catalytic activity
Cluster-12329.61956	0.25	0.0127	0.18	0.0403	catalytic activity
Cluster-12329.70108	8.16	0.0001	8.62	0.0000	catalytic activity
Cluster-12329.68563	0.29	0.0206	0.39	0.0266	carbohydrate metabolic process
Cluster-12329.29591	2.03	0.0030	4.49	0.0162	carbohydrate metabolic process
Cluster-12329.34933	2.81	0.0033	3.02	0.0219	carbohydrate metabolic process
Cluster-12329.43587	0.28	0.0001	0.23	0.0000	carbohydrate metabolic process
Cluster-12329.48578	0.44	0.0371	0.40	0.0496	carbohydrate metabolic process
Cluster-12329.51456	2.57	0.0170	2.88	0.0067	carbohydrate metabolic process
Cluster-12329.51985	0.36	0.0183	0.17	0.0317	carbohydrate metabolic process
Cluster-12329.53243	0.36	0.0060	0.28	0.0118	carbohydrate metabolic process
Cluster-12329.59871	0.19	0.0206	0.20	0.0212	carbohydrate metabolic process
Cluster-12329.61460	0.34	0.0023	0.23	0.0003	carbohydrate metabolic process
Cluster-12329.69576	0.38	0.0021	0.46	0.0048	carbohydrate metabolic process
Cluster-12329.48513	3.38	0.0004	2.95	0.0020	calcium ion binding
Cluster-12329.63459	2.17	0.0344	2.05	0.0229	calcium ion binding
Cluster-12329.36041	0.39	0.0035	0.43	0.0054	caffeoyl-CoA O-methyltransferase
Cluster-12329.21254	0.46	0.0232	0.26	0.0102	biosynthetic process
Cluster-12329.19475	2.32	0.0191	2.05	0.0111	Biological Process:cell redox homeostasis
Cluster-12329.49557	2.16	0.0114	2.39	0.0347	ATP-dependent RNA helicase DOB1
Cluster-12329.44150	2.20	0.0013	2.33	0.0100	ATP-dependent RNA helicase DBP3
Cluster-12329.36548	0.34	0.0369	0.41	0.0383	ATP binding
Cluster-12329.39910	0.18	0.0089	0.13	0.0067	ATP binding
Cluster-12329.65472	0.47	0.0172	0.37	0.0052	ATP binding
Cluster-8220.0	0.34	0.0276	0.13	0.0291	aspartic-type endopeptidase activity
Cluster-12329.33762	0.22	0.0024	0.17	0.0022	aspartic-type endopeptidase activity
Cluster-12329.47560	2.51	0.0004	3.62	0.0473	aspartic-type endopeptidase activity
Cluster-12329.47580	0.49	0.0080	0.22	0.0013	aspartic-type endopeptidase activity
Cluster-12329.49924	0.31	0.0087	0.41	0.0116	aspartic-type endopeptidase activity
Cluster-12329.58062	0.43	0.0273	0.32	0.0446	aspartic-type endopeptidase activity
Cluster-12329.48665	2.31	0.0312	2.12	0.0102	asparagine synthase (glutamine-hydrolysing)
Cluster-12329.64984	0.40	0.0018	0.18	0.0002	amidase activity
Cluster-12329.47418	0.42	0.0004	0.41	0.0060	actin, other eukaryote
Cluster-12329.43923	0.45	0.0167	0.37	0.0141	actin binding
Cluster-12329.51553	0.37	0.0031	0.21	0.0003	actin beta/gamma 1
Cluster-12329.46649	0.37	0.0107	0.25	0.0048	acid phosphatase activity
Cluster-12329.54954	3.38	0.0006	2.63	0.0320	acid phosphatase activity
Cluster-12329.76839	0.35	0.0004	0.29	0.0012	acid phosphatase activity
Cluster-12329.26903	2.96	0.0006	2.77	0.0006	6-phosphofructokinase activity
Cluster-12329.48062	0.32	0.0051	0.27	0.0039	4-coumarate--CoA ligase
Cluster-12329.70084	2.63	0.0004	2.11	0.0187	4-alpha-glucanotransferase activity
Cluster-12329.52214	0.49	0.0342	0.25	0.0138	3-phosphoshikimate 1-carboxyvinyltransferase
Cluster-12329.33319	2.60	0.0164	2.92	0.0130	3-hydroxyisobutyryl-CoA hydrolase activity
Cluster-17679.0	2.02	0.0001	2.45	0.0000	1-pyrroline-5-carboxylate dehydrogenase
Cluster-10701.1	3.83	0.0172	5.11	0.0004	--

Cluster-12329.45692	0.45	0.0033	0.41	0.0028	--
Cluster-12329.12	0.43	0.0025	0.32	0.0086	--
Cluster-12329.15521	0.46	0.0293	0.38	0.0317	--
Cluster-12329.16465	2.81	0.0373	5.13	0.0242	--
Cluster-12329.19760	4.98	0.0022	6.54	0.0041	--
Cluster-12329.20993	3.82	0.0278	5.00	0.0146	--
Cluster-12329.21425	0.47	0.0023	0.26	0.0001	--
Cluster-12329.22132	2.83	0.0051	2.06	0.0416	--
Cluster-12329.24467	5.39	0.0001	6.15	0.0240	--
Cluster-12329.26112	2.72	0.0034	2.15	0.0499	--
Cluster-12329.30725	0.39	0.0245	0.16	0.0296	--
Cluster-12329.30855	0.33	0.0192	0.39	0.0190	--
Cluster-12329.31252	2.46	0.0419	15.22	0.0079	--
Cluster-12329.32559	2.00	0.0241	2.87	0.0202	--
Cluster-12329.33447	2.36	0.0060	2.27	0.0161	--
Cluster-12329.34089	2.03	0.0055	2.41	0.0016	--
Cluster-12329.34631	0.27	0.0001	0.18	0.0000	--
Cluster-12329.37085	0.37	0.0226	0.33	0.0384	--
Cluster-12329.37995	0.11	0.0119	0.24	0.0145	--
Cluster-12329.40468	0.46	0.0318	0.24	0.0156	--
Cluster-12329.40963	0.45	0.0022	0.21	0.0016	--
Cluster-12329.40989	2.99	0.0472	4.62	0.0085	--
Cluster-12329.41906	7.56	0.0001	6.54	0.0353	--
Cluster-12329.41956	6.01	0.0008	9.31	0.0266	--
Cluster-12329.42024	0.50	0.0204	0.42	0.0345	--
Cluster-12329.42053	0.34	0.0113	0.21	0.0041	--
Cluster-12329.42685	2.09	0.0018	2.25	0.0012	--
Cluster-12329.44022	2.01	0.0371	2.89	0.0033	--
Cluster-12329.44215	7.51	0.0198	5.86	0.0018	--
Cluster-12329.44647	0.41	0.0328	0.33	0.0242	--
Cluster-12329.44648	0.39	0.0017	0.24	0.0010	--
Cluster-12329.44648	0.29	0.0018	0.32	0.0020	--
Cluster-12329.44802	0.41	0.0005	0.40	0.0044	--
Cluster-12329.44810	3.53	0.0073	3.62	0.0070	--
Cluster-12329.45397	2.46	0.0003	2.44	0.0000	--
Cluster-12329.45476	2.10	0.0290	3.18	0.0110	--
Cluster-12329.45721	2.84	0.0371	4.08	0.0124	--
Cluster-12329.45893	0.41	0.0104	0.32	0.0171	--
Cluster-12329.46781	0.42	0.0123	0.26	0.0052	--
Cluster-12329.48367	0.46	0.0180	0.45	0.0181	--
Cluster-12329.51534	0.30	0.0220	0.28	0.0188	--
Cluster-12329.51806	0.37	0.0030	0.16	0.0009	--
Cluster-12329.53646	0.25	0.0078	0.13	0.0200	--
Cluster-12329.53863	0.48	0.0359	0.36	0.0195	--
Cluster-12329.54583	3.24	0.0014	3.14	0.0027	--
Cluster-12329.54669	0.34	0.0011	0.39	0.0005	--
Cluster-12329.55286	0.19	0.0317	0.29	0.0432	--
Cluster-12329.55503	2.30	0.0049	2.24	0.0148	--
Cluster-12329.55504	4.14	0.0401	3.92	0.0005	--
Cluster-12329.55770	2.80	0.0096	3.73	0.0397	--
Cluster-12329.55856	2.20	0.0263	2.48	0.0195	--

Cluster-12329.56671	0.33	0.0117	0.36	0.0185	--
Cluster-12329.63149	0.19	0.0006	0.09	0.0001	--
Cluster-12329.63577	3.94	0.0436	3.01	0.0429	--
Cluster-12329.64664	2.64	0.0467	2.82	0.0048	--
Cluster-12329.65108	2.83	0.0018	3.37	0.0156	--
Cluster-12329.66452	3.06	0.0005	3.11	0.0014	--
Cluster-12329.68807	2.60	0.0273	2.80	0.0024	--
Cluster-12329.69143	4.48	0.0015	4.44	0.0120	--
Cluster-12329.75135	7.23	0.0051	6.71	0.0198	--
Cluster-2111.0	0.47	0.0487	0.26	0.0296	--

Table S5. Primers used in real-time quantitative reverse transcription PCR (qRT-PCR) experiment.

Gene	Description	Sequence (5′ to 3′)	Length(bp)
Cluster-12329.57977	protein kinase activity	F: CTCCTCACGCTTCATGGGTT<br>R: TACGAGGTTGGGAGCCTTCT	280
Cluster-12329.43859		F:CGCCGACGAGATCTTCCAAA<br>R:AGAATTGGAGGCAGTCCCAC	269
Cluster-12329.60427	Gamma-glutamyl kinase	F:AGGGGAGGTGACGAAGAGG<br>R:CTCCTCGACGCCTGCAAC	170
Cluster-12329.60324	Phosphoglycerate mutase	F:TCCTGAAGCAGAAGCAGAGC<br>R:GATCTCTCCAGCAGTCCGTC	104
Cluster-12329.70108	Thiamine pyrophosphate-requiring enzyme	F:GAGGGGAAGTGGAGGGAAGA<br>R:CCCCCAAAACAGAAATGGCG	170
Cluster-12329.82252	Glyceraldehyde 3-phosphate dehydrogenase	F:CGCAGCTGAGTACAGATCGA<br>R:GGCTTTGATTTGGTTCCCCG	255
Cluster-12329.35240	hydrolase activity	F:AGCTCACACAGGTTGTAGGC<br>R:CCACCGCCGATTGGAATAGA	158
Cluster-12329.36165	GDP-mannose pyrophosphorylase	F:TGAAGGAACAGAGCTGGCAG<br>R:GGATGACACGGTGGAGGATC	216
Cluster-12329.29020	sucrose synthase activity	F:AGGTTTCCTCTGTTCTGGCT<br>R:GGCTCTCGTACTGTCCAACA	263
Cluster-12329.62359	Eukaryotic-type DNA primase	F:GCTCTCGACGCCCAATGTAT<br>R:GCAGTACGCACAGTCGATCT	251
Cluster-12329.59605	NADP+-dependent malic enzyme	F:AGATCCTCACGCTGCACTTC<br>R:ACCAGAACAGGCTGAGCATC	227
Cluster-12329.54954	catalytic activity	F:TCACCCCGCTTCATTCTTCT<br>R:AGCCTTGCACGGTACCATAG	257
Cluster-12329.59603	NAD binding	F:GTGGAAGGTCGTGAAGCTGA<br>R:CCTCCACCACTTCACTCACC	116
actin	Avena sativa actin (ACT) mRNA, partial cds	F: CCAATCGTGAGAAGATGACCC<br>R: CACCATCACCAGAATCCAACA	135
